# Downregulation of ciRNA‐ *Kat6b* in dorsal spinal horn is required for neuropathic pain by regulating *Kcnk1* in miRNA‐26a‐dependent manner

**DOI:** 10.1111/cns.14235

**Published:** 2023-05-05

**Authors:** Ling Xie, Ming Zhang, Qiaoqiao Liu, Runa Wei, Menglan Sun, Qi Zhang, Lingyun Hao, Zhouya Xue, Qihui Wang, Li Yang, Hongjun Wang, Zhiqiang Pan

**Affiliations:** ^1^ Jiangsu Province Key Laboratory of Anesthesiology Xuzhou Medical University Xuzhou China; ^2^ Jiangsu Province Key Laboratory of Anesthesia and Analgesia Application Technology Xuzhou Medical University Xuzhou China; ^3^ NMPA Key Laboratory for Research and Evaluation of Narcotic and Psychotropic Drugs Xuzhou China; ^4^ Department of Anesthesiology The Obstetrics and Gynecology Hospital of Fudan University Shanghai China; ^5^ Department of Anesthesiology The Yancheng First People's Hospital Affiliated to Xuzhou Medical University Yancheng China

**Keywords:** circRNA‐*Kat6b*, *Kcnk1*, miR‐26a, neuropathic pain

## Abstract

**Aims:**

Nerve injury‐induced maladaptive changes in gene expression in the spinal neurons are essential for neuropathic pain genesis. Circular RNAs (ciRNA) are emerging as key regulators of gene expression. Here, we identified a nervous‐system‐tissues‐specific *ciRNA‐Kat6* with conservation in humans and mice. We aimed to investigate whether and how spinal dorsal horn *ciRNA‐Kat6b* participates in neuropathic pain.

**Methods:**

Unilateral sciatic nerve chronic constrictive injury (CCI) surgery was used to prepare the neuropathic pain model. The differentially expressed ciRNAs were obtained by RNA‐Sequencing. The identification of nervous‐system‐tissues specificity of *ciRNA‐Kat6b* and the measurement of *ciRNA‐Kat6b* and *microRNA‐26a* (*miRNA‐26a*) expression level were carried out by quantitative RT‐PCR. The *ciRNA‐Kat6b* that targets *miRNA‐26a* and *miRNA‐26a that* targets *Kcnk1* were predicted by bioinformatics analysis and verified by in vitro *luciferase reports* test and in vivo experiments including Western‐blot, immunofluorescence, and RNA–RNA immunoprecipitation. The correlation between neuropathic pain and *ciRNA‐Kat6b*, *miRNA‐26a*, or *Kcnk1* was examined by the hypersensitivity response to heat and mechanical stimulus.

**Results:**

Peripheral nerve injury downregulated *ciRNA‐Kat6b* in the dorsal spinal horn of male mice. Rescuing this downregulation blocked nerve injury‐induced increase of *miRNA‐26a*, reversed the *miRNA‐26a*‐triggered decrease of potassium channel *Kcnk1*, a key neuropathic pain player, in the dorsal horn, and alleviates CCI‐induced pain hypersensitivities. On the contrary, mimicking this downregulation increased the *miRNA‐26a* level and decreased *Kcnk1* in the spinal cord, resulting in neuropathic pain‐like syndrome in naïve mice. Mechanistically, the downregulation of *ciRNA‐Kat6b* reduced the accounts of *miRNA‐26a* binding to *ciRNA‐Kat6b*, and elevated the binding accounts of *miRNA‐26a* to the 3′ untranslated region of *Kcnk1* mRNA and degeneration of *Kcnk1* mRNA, triggering in the reduction of KCNK1 protein in the dorsal horn of neuropathic pain mice.

**Conclusion:**

The *ciRNA‐Kat6b*/*miRNA‐26a/Kcnk1* pathway in dorsal horn neurons regulates the development and maintenance of neuropathic pain, *ciRNA‐Kat6b* may be a potential new target for analgesic and treatment strategies.

## Graphical abstract

The *ciRNA‐Kat6b/miRNA‐26a/Kcnk1* pathway in dorsal horn neurons regulates the development and maintenance of neuropathic pain which may be a potential therapeutic target.

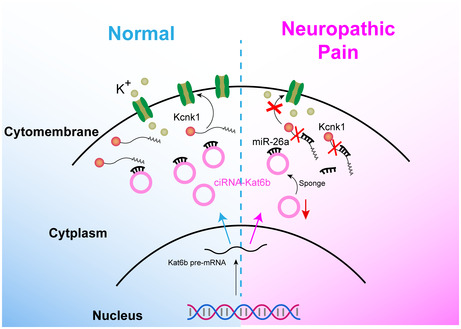

## 1 INTRODUCTION

Nerve injury‐induced neuropathic pain is a chronic and refractory disease, therefore affecting the quality of life of many patients. It is estimated that neuropathic pain in the general population is to have a prevalence ranging between 3% and 17%.^1^ In the United States, over 600 billion dollars per year is spent on healthcare costs related to neuropathic pain management.^2^ However, as current medications such as opioids and nonsteroidal anti‐inflammatory drugs are ineffective or have severe side effects in most neuropathic pain patients, the therapeutic effect is limited.^3^ Neuropathic pain is characterized by abnormal hypersensitivity to stimuli (hyperalgesia) and nociceptive responses to non‐noxious stimuli (allodynia). Central (e.g., spinal cord dorsal) sensitization is thought to be a vital role in pain hypersensitivity. These abnormal spinal sensitization activities are responsible for peripheral nerve injury‐induced maladaptive changes in such pain‐associated genes as an ion channel, receptor, and intercellular signal molecular in the spinal cord.^4^, ^5^, ^6^, ^7^, ^8^, ^9^ Exploring the regulation mechanism of dysfunctional spinal genes may provide a new avenue for neuropathic pain management.

Circular RNAs (circRNAs), as a kind of novel identified non‐coding RNAs, have attracted great attention due to their potent and multifunction in the regulation of gene expression.^10^, ^11^ A large number of circRNAs are dysregulated in the spinal cord following peripheral nerve injury,^12^, ^13^ but the molecular mechanism underlying neuropathic pain is still poorly understood. We identified a nervous system‐specifically expressed circRNA — ciRNA‐*Kat6b* — is highly enriched in the spinal cord and decreases the expression in the spinal cord after peripheral nerve injury. But it remains unclear the mechanism of ciRNA‐*Kat6b* involved in neuropathic pain.

Potassium channels control the excitability of spinal neurons, therefore, become a critical player in the occurrence of pain hypersensitivity.^14^ KCNK1 belongs to the two‐pore domain background potassium (K2P) channel family and is the first identified member in this family. *Kcnk1* is enriched in the peripheral and central nervous systems such as dorsal root ganglion, trigeminal nerve, and spinal cord,^15^, ^16^ peripheral nerve injury decreases the level of *Kcnk1* in mouse DRG.^17^ Rescuing this decrease alleviates nerve injury‐induced mechanical, thermal, and cold pain hypersensitivities. *Kcnk1* has become a potential modulation factor in the development and maintenance of neuropathic pain.^17^ Here, we demonstrated that the decreased of ciRNA‐*Kat6b* contributes to development and maintenance of nerve injury‐induced neuropathic pain by regulating *microRNA‐26a (miR‐26a)*‐triggered *Kcnk1* in spinal cord neurons. *ciRNA‐Kat6b* may be a critical player in neuropathic pain.

## 2 MATERIALS AND METHODS

### 2.1 Animals and pain model

Adult male Kunming mice (20–25 g) were used in this study. All animal procedures were approved by the animal care committee of Xuzhou Medical University (Xuzhou, China). All animals were kept at 25°C and 40% humidity under a 12:12 h light/dark cycle and with ad libitum access to food and water. Chronic inflammatory pain was induced by subcutaneous administration of CFA (40 μl; F5881; Sigma‐Aldrich) into the plantar surface of the left hind paw. The unilateral sciatic nerve chronic constrictive injury (CCI) model was performed as described previously.^4^ All mice were maintained in a warm environment until they recovered from anesthesia.

### 2.2 Behavioral tests

Before the behavior testing, the locomotor function was tested according to the previous method.^9^ Thermal hyperalgesia and mechanical allodynia were measured, respectively, as previously described.^8^ Briefly, thermal nociceptive behavior was assessed by measuring mouse paw‐withdrawal latency in response to a thermal stimulus with an analgesia meter (IITC Model336 Analgesia Meter, Series 8; IITC Life Science). The time required for the stimulus to elicit withdrawal of the hind paw was recorded. Von Frey (Stoelting) filaments that produce different forces were applied to detect mechanical allodynia. Starting with a 0.16 g and ending with a 6.0 g filament. In the absence of a paw withdrawal response, a stronger stimulus was presented; when paw withdrawal occurred, the next weaker stimulus was chosen. The optimal threshold filaments were presented five times, respectively, at 5 min intervals. All behavioral tests were performed in a double‐blind trial fashion in this study.

### 2.3 Spinal and DRG tissue collection

Mice were anesthetized with isoflurane and the spinal cord within the lumbar segments (L3–L5) was removed rapidly. The DRG of the spinal lumbar segment (L3/4 DRGs) was extracted. All operations were performed on the ice box and put into snap‐frozen in liquid nitrogen, and stored at −80°C.

### 2.4 RNA, circRNA, miRNA, and qRT‐PCR

Total RNA was isolated with a Trizol reagent (15596‐026; Invitrogen) to generate cDNA templates by reverse transcription reactions with random primers and reverse transcriptase M‐MLV (2641A; Takara Bio) at 42°C for 60 min. cDNA products were used as templates to detect gene mRNA (*Kat6b* F: 5′‐AGAAGAAAAGGGGTCGTAAACG‐3′; R: 5′‐GTGGGAATGCTTTCCTCAGAA‐3′; *Kcnk1* F: 5′‐GAGGAGCTGCCTTATGAGGAC‐3′; R: 5′‐TCCCAATTCCAATTTCCCGAG‐3′; GAPDH F: 5′ ‐AGGTCGGTGTGAACGGATTTG‐3′, R: 5′‐TGTAGACCATGTAGTTGAGGTCA‐3′; U6 F: 5′‐CTCGCTTCGGCAGCACATATACT‐3′, R: 5′‐ACGCTTCACGAATTTGCGTGTC‐3′; Human β‐Tubulin F: 5′‐GGCCAAGGGTCACTACACG‐3′, R: 5′‐GCA GTCGCAGTTTTCACACTC‐3′), or circRNA (ciRNA‐*Kat6b* F: 5′‐GTGCTTTTCCGTCCTCACTC‐3′, R: 5′‐ACACAACCTGCACATTCCAA‐3′; Human ciRNA‐*Kat6b* F: 5′‐GTGCATTCCCATCCTCGCTC‐3′, R: 5′‐ACAGAACTTGCACATTTGAA‐3′) via qRT‐PCR with SYBR Premix ExTaqII (RR820A; Takara Bio) according to the manufacturer's instructions. MiRNA was reversely transcribed at 16°C for 30 min, and 37°C for 30 min using specific primer 26aRT (5′‐TTAACTGGATACGAAGGGTCCGAACACCGGTCGTATCCAGTTAAAGCCTATC‐3′). qRT‐PCR was performed using primer pairs (miRNA‐26a F: 5′‐TGCGGTTCAAGTAATCCAGGA‐3′, R: 5′‐TACGAAGGGTCCGAACAC‐3′). For RNase R treatment, 5 μg of RNA was incubated with 4 U/μg of RNase R (Epicenter) for 20 min at 37°C and purified by phenol–chloroform extraction. GAPDH and U6 as internal control. The expression levels of the target genes were quantified relative to *Gapdh* or U6 expression (cycle threshold [Ct]) using the 2^−∆∆CT^ methods.

### 2.5 Spinal neuron cell culture

The culture of spinal neuron cells was carried out described previously.^8^ Briefly, 3 to 4 days‐old mice under deep anesthesia were decapitated and the lumbar L3 to L5 segments of the dorsal spinal cord were collected. After enzymatical digestion with papain and mechanical dissociation, the homogenate was centrifugated for 5 min at 500 rpm. After centrifugated, the supernatant was removed and replaced with 5 mL of culture medium, the composition of which was as follows: MEM‐(Invitrogen), FCS (5% v/v; Invitrogen), heat‐inactivated horse serum (5% v/v; Invitrogen), penicillin, and streptomycin (50 IU/mL for each; Invitrogen), transferrin (10 mg/mL; Sigma‐Aldrich), insulin (5 mg/mL; Sigma‐Aldrich), putrescine (100 nM; Sigma‐Aldrich), and progesterone (20 nM; Sigma‐Aldrich). After trituration with a fire‐polished Pasteur pipette, the cells were plated on plastic culture dishes. Cultures were maintained in a water‐saturated atmosphere (95% air, 5% CO_2_) at 37°C until used (10–15 days). Two days after the cells were seeded, cytosine arabinoside was added to the culture medium for 24 h to reduce glial proliferation.

### 2.6 Spinal astrocytes and microglia cultures

The isolation of spinal astrocytes and microglia cells was performed as described previously with few modifications.^8^ Briefly, 3 to 4 days‐old mice under deep anesthesia were decapitated and the lumbar L3 to L5 segments of the dorsal spinal cord were collected, then tissues were digested enzymatically for 45 min at 37°C with trypsin and stopped by mixing the DMEM with 10% FBS. After centrifuging at 1000 g for the 30 s, the supernatant was removed and washed three times with DMEM. Suspended the cells by DMEM with 20% FBS and filtered with 200 mesh sieve. The cells were plated into a 24‐well plate and cultured at 37°C for 1 week. To harvest the astrocyte the mixed cell were shaken overnight and discarded the supernatant. To harvest microglia cells, the mixed cells were shaken for 4 h, and collected the supernatant cell to a new plate for culture.

### 2.7 Single‐cell RT‐PCR

Single‐cell RT‐PCR for spinal neurons was performed as described previously with few modifications.^8^ The primers are shown as follows: ciRNA‐*Kat6b*: outF, 5′‐GTGCTTTTCCGTCCTCACTC‐3′; outR, 5′‐ACAACCTGCACATTCCAA‐3′; inF. 5′‐AGCCTTCTACCCCATGAGAAA‐3′; inR, 5′‐CAACAGCATAGGGACAACCAT‐3′. MiRNA‐26a outF was the same as the miRNA‐26a F primer mentioned above; out R, 5′‐TTAACTGGATACGAAGGGT‐3′. MiRNA‐26a inF, 5′‐GGCTGGTGGAGTTAACTGG‐3′, and inR, 5′‐AAGGGTCCGAACACACCG‐3′. NeuN: outF, 5′‐AGACAGACAACCAGCAACTC‐3′; outR, 5′‐CTGTTCCTACCACAGGGTTTAG‐3′. inF, 5′‐ACGATCGTAGAGGGACG‐3′; inR, 5′‐TTGGCATAGGGTTCCCAGG‐3′. Gapdh: outF, 5′‐AGGTTCATCAGGTAAACTCAG‐3′; outR, 5′‐ACCAGTAGACTCCACGACAT‐3′. inF, 5′‐ACCAGGGCTGCCATTTGCA; inR, 5′‐CTCGCTCCTGGAAGATGGTG‐3′. Gapdh was used as the reference gene.

### 2.8 Plasmid construction

All constructs were produced by the use of standard molecular methods and confirmed by DNA sequencing. The overexpressed fragment was prepared by PCR, Lenti‐ciRNA‐*Kat6b* F: 5′‐ATAACGCGTTCTAGGGGTTGAACTCAGGT‐3′ (*Ml*uI), R: 5′‐ATAGAATTCCAAGGCACAAACAACTGATCTT‐3′ (*Eco*RI); Lenti‐miRNA‐26a F: 5′‐ATAACGCGTTCTTCACACCACGTGGCCA‐3′ (*Ml*uI), R: 5′‐ATAGAATTCCTGCAGGACCTGCTTTGCT‐3′ (*Eco*RI); Lenti‐*Kcnk1* F: 5′‐ATAACGCGTGCCACCATGCTGCAGTCCCTGGCCG‐3′ (*Mlu*I), R: 5′‐ATAGAATTCTCAGTGGTCTGCAGAGCCA‐3′ (*Eco*RI). The lentivirus for knockdown of the expression of *ciRNA‐Kat6b* and *miRNA‐26a* are LV‐ciRNA‐*Kat6b*‐shRNA F: 5′‐CGCGTCCCCAGGAAACCAAGACCTGGTCTTCAAGAGAGACCAGGTCTTGGTTTCCTTTTTGGAAAT‐3′ (*Mlu*1), R: 5′‐CGATTTCCAAAAAGGAAACCAAGACCTGGTCTCTCTTGAAGACCAGGTCTTGGTTCCTGGGGA‐3′ (*Cla*I); LV‐miRNA‐26a Ih, F: 5′‐CGCGTAGCCTATCCTGGATTACTTGAATTCAAGAGATTCAAGTAATCCAGGATAGGCTTTTTTGGAAAT‐3′ (*Mlu*1), R: 5′‐CGATTTCCAAAAAAGCCTATCCTGGATTACTTGAATCTCTTGAATTCAAGTAATCCAGGATAGGCTA‐3′ (*Cla*I). These amplified fragments and PWPXL vector were digested by corresponding double restriction endonucleases, and then ligated with T4 ligase.

### 2.9 Lentivirus production and verification

The constructed core plasmid (8 μg) and two envelope plasmids, PSPAX2 (6 μg), and PMD2G (2 μg) were co‐transfected into HEK293T cells in a 6‐well plate according to the manufacturer's instructions of Lipofectamine 2000 (11668‐027, Invitrogen). The supernatant was collected at 48 h after transfection and concentrated by using a Centricon Plus‐70 filter unit (UFC910096, Millipore). Lentivirus with titers 10^8^ TU/mL was used in the experiment. The virus was validated in vivo and in vitro as in previous study.^4^ In in vitro test, 20 μL lentivirus were added in a 24‐well plate containing 1 × 10^5^ HEK293T cells and DMEM without FBS; after 24 h, the transfection medium was replaced with 500 μL fresh complete medium containing 10% FBS; cells were collected at 48 h after culture and then detected the abundance of the target gene. In in vivo test, daily intrathecal injections of lentivirus or vector (1 μL) were performed for 2 consecutive days in naïve or pain mice and then collected samples day 3 after the first injection to detective the expression of the target gene.

### 2.10 SiRNA, mimics and inhibitor

The siRNA, mimics, and inhibitor were used for intrathecal injection as described previously.^5^ In brief, holding the mouse by the pelvic girdle and inserting a 30‐gauge needle attached to a 25 μL microsyringe between L5 and L6 vertebrae. When the needle was inserted into the subarachnoid space, the mouse tail was found to swing slightly. Injections of 5 μL of 20 μM siRNAs, mimics, and inhibitors. The expression efficiency of *cirRNA‐Kat6b*‐siRNA (5′‐AGGAAACCAAGACCUGGUC‐3′), *miRNA‐26a* mimics (5′‐UUCAAGUAAUCCAGGAUAGGCU‐3′), *miRNA‐26a* inhibitor (5′‐AGCCUTU CCUGGTUUTCUUGAA‐3′), *Kcnk1*‐siRNA (604S, 5′‐GCUGGAGGCCAGCAAUUAUTT‐3′, 604AS 5′‐AUAAUUGCUGGCCUCCAGCTT‐3′. 843S 5′‐GCAGACCAGUCCUCUACUUTT‐3′; 843AS, 5′‐AAGUAGAGGACUGGUCUGCTT‐3′) were confirmed with qRT‐PCR or Western‐Blot from samples of the ipsilateral dorsal spinal cord at 72 h after the last injection. Animals receiving intrathecal injections of scrambled control were used as control groups.

### 2.11 Construction of reporter vector

Construction of *circRNA‐Kat6b* sponging *miRNA‐26a* reporter: a fragment of *circRNA‐Kat6b* containing the bound sequence by *miRNA‐26a* was amplified from mouse genomic DNA using a pair of primers (F: 5′‐CGCTCGAGAGGACGGAGCCCAGGCAG‐3′, R: 5′‐CGACGCGTAACCAATCAGCTGGCGGGA‐3′). The PCR products and pGL6 (5:1) were mixed, digested with *Xho*l and *Mlu*I, then ligated with T4 ligase. The ligation product is named the wild type reporter (pGL6‐wt‐*ciR‐Kat6b*). Then, pGL6‐wt‐ciR‐*Kat6b* was used as a PCR template to amplify the mutated fragment using a pair of primers with mutation‐matched sites (MF: 5′‐AGACATAGGATGGCGTATTTCATCGGGTTAAC‐3′, MR: 5′‐AAATACGCCATCCTATGTCTGGGTCCTTATATG‐3′). PCR products were ligated using a Gibson kit (E5510S), namely the mutated type reporter (pGL6‐mut‐*ciR‐Kat6b*).

To construct the reporter of *miRNA‐26a* targeting *Kcnk1*, the matched fragment of *Kcnk1* 3′‐untranslated region (3′‐UTR) was amplified with a pair of primers (wild‐type F: 5′‐ATACTCGAGGAGCCTTGACTGCATCCATTT‐3′, R: 5′‐ATAGCGGCCGCTGTTGCTGCTTAGCTGTCGG‐3′). The PCR products and psiCHECK2 were mixed, digested with *Xho*l and *Not*I, and ligated with T4 ligase, namely wild‐type reporter (CHK‐wt‐*Kcnk1*). The mutation reporter was obtained by amplifying the fragment with a CHK‐wt‐*Kcnk1* template and a pair of primers (mutated‐type F: 5′‐TTCAAGCAACAGTGTACTGCAGTAGGCG‐3′, R: 5′‐AGTACACTGTTGCTTGAATTCGTCCCCAGTGTA‐3′), and ligating the PCR fragment using Gibson kit, namely mutated type reporter (CHK‐wt‐*Kcnk1*).

### 2.12 Luciferase reporter assay

HEK293T cell was cultured in DMEM with 10% FBS. The reporter plasmid (50 ng) and *miR‐26a* mimics (80 ng) or its inhibitor (80 ng) were co‐transfected into HEK293T cells using Lipofectamine 2000 (11668‐027, Invitrogen) in a 24‐well plate. Cell lysis and fluorescence intensity were performed using dual luciferase kits (E1910, Promega) at 36–48 h after transfection according to the manufacturer's instruction.

### 2.13 Western blot analysis

RIPA was used to lyse cells or tissues to extract proteins. 20–50 μg of Protein was separated with 10% SDS‐PAGE gel, transferred onto a PVDF membrane, and incubated with antibody against KCNK1 (1:1000 arb390214 biorbyt) or GAPDH (1:5000 AP0066 bioworld) and H3 (1:2000 ab1791 abcam). Incubated in peroxidase‐conjugated goat anti‐rabbit IgG secondary antibody (1:1000; VA001, Vicmed) and visualized using chemiluminescence detection (72,091, Sigma‐Aldrich).

### 2.14 Immunofluorescence and fluorescence in suit hybridization (FISH)

To prepare complementary RNA (antisense cRNA) probes of mouse *ciRNA‐Kat6b*, a PCR product was amplified using mouse cDNA with a pair of primers including the T7 promoter at the 3′ end (F, 5′‐GTGCTTTTCCGTCCTCACTC‐3′; T7R, 5′‐TAATA CGACTCACTATAGGGACACAACCTGCACATTCCAA‐3′). Sense cRNA probes of *ciRNA‐Kat6b* were prepared from a PCR product amplified using mouse cDNA with a pair of primers including the T7 promoter at the 5′ end (T7F, 5′‐TAATACGACTCACTATAGGGGTGCTTTTCCGTCCTCACTC‐3′; R, 5′‐ACACAACCTGCACATTCCAA‐3′). The sequence was identified using DNA sequencing. After PCR purification, a riboprobe was generated through in vitro transcription and labeled with digoxigenin‐dUTP according to the manufacturer's instructions (Roche Diagnostics, Indianapolis, IN) at 37°C for 2 h. The probes were purified using Micro Bio‐Spin™ 30 Chromatography Column (Bio‐Rad). FISH was carried out as described previously with minor modification.^18^ After being treated with proteinase K for 15 min, two sets of sections were pre‐hybridized for 1.5 h at 65°C and hybridized with digoxigenin‐dUTP‐labeled antisense and sense cRNA probes, respectively, at 65°C overnight. After being blocked, the sections were incubated with alkaline phosphatase‐conjugated sheep anti‐digoxigenin (1:500, Roche) and NeuN antibody (1:1000; MAB2300, Millipore) overnight at 4°C. After being washed in 1× PBST, TNT buffer, and detection buffer, respectively, the fluorescent signals were developed by incubation with Fast‐Red dye and AlexaFluor‐conjugated secondary antibodies (Cell Signaling Technology).

### 2.15 RNA–RNA in vivo precipitation (RRIP)

According to previous described,^8^ with modification, biotin‐labeled wild or mutation *miRNA‐26a* probe (Wild bio‐26a, 5′‐TTCAAGTAATCCAGGATAGGCT‐Bio‐3′; Mutated bio‐26a, 5′‐ATCCCTTAAAGGTTCTCAGGC‐Bio‐3′) or biotin‐labeled wild or mutation *ciRNA‐Kat6b* probe (Wild Bio‐K6b, 5′‐CATCTTTAGGAAACCAAGACCTGGTCTTTCTCATGGGGT‐Bio‐3′; Mutated Bio‐K6b, 5′‐ACCCCATGAGAAAGACCAGGTCTTGGTTTCCTAAAGATG‐Bio‐3′) were used to perform the RRIP experiment assay. The spinal cord was harvested 24 h after intrathecal injection of Bio‐26a (5 μL, 20 μM) and fixed by 2.5% formaldehyde for 10 min, lysed, and sonicated. After centrifugation, 50 μL of the supernatant was retained as input, and the remaining part was incubated with Dyna‐beads M‐280 Streptavidin (11205D, Thermo Fisher Scientific) mixture overnight at 4°C. The next day, a beads‐probes‐RNAs mixture was washed and incubated with 200 μL lysis buffer and proteinase K to reverse the formaldehyde crosslinking. Finally, the mixture was added with TRizol for RNA extraction and detection.

### 2.16 Statistical analysis

All data were first tested for normality using a Shapiro–Wilk test normality test by Prism GraphPad 8.0. The data were presented as Means ± SEM. One‐way or two‐way ANOVA or paired or unpaired Student's *t*‐test were used to statistically analyzed. When ANOVA showed a significant difference, pairwise comparisons between means were tested by the post hoc Tukey method. Statistical analyses were performed with Prism (GraphPad 8.0). *p* < 0.05 was considered statistically significant in all analyses.

## 3 RESULTS

### 3.1 Identification of spinal *ciRNA‐Kat6b* and its specificity in the nervous system

**FIGURE 1:**
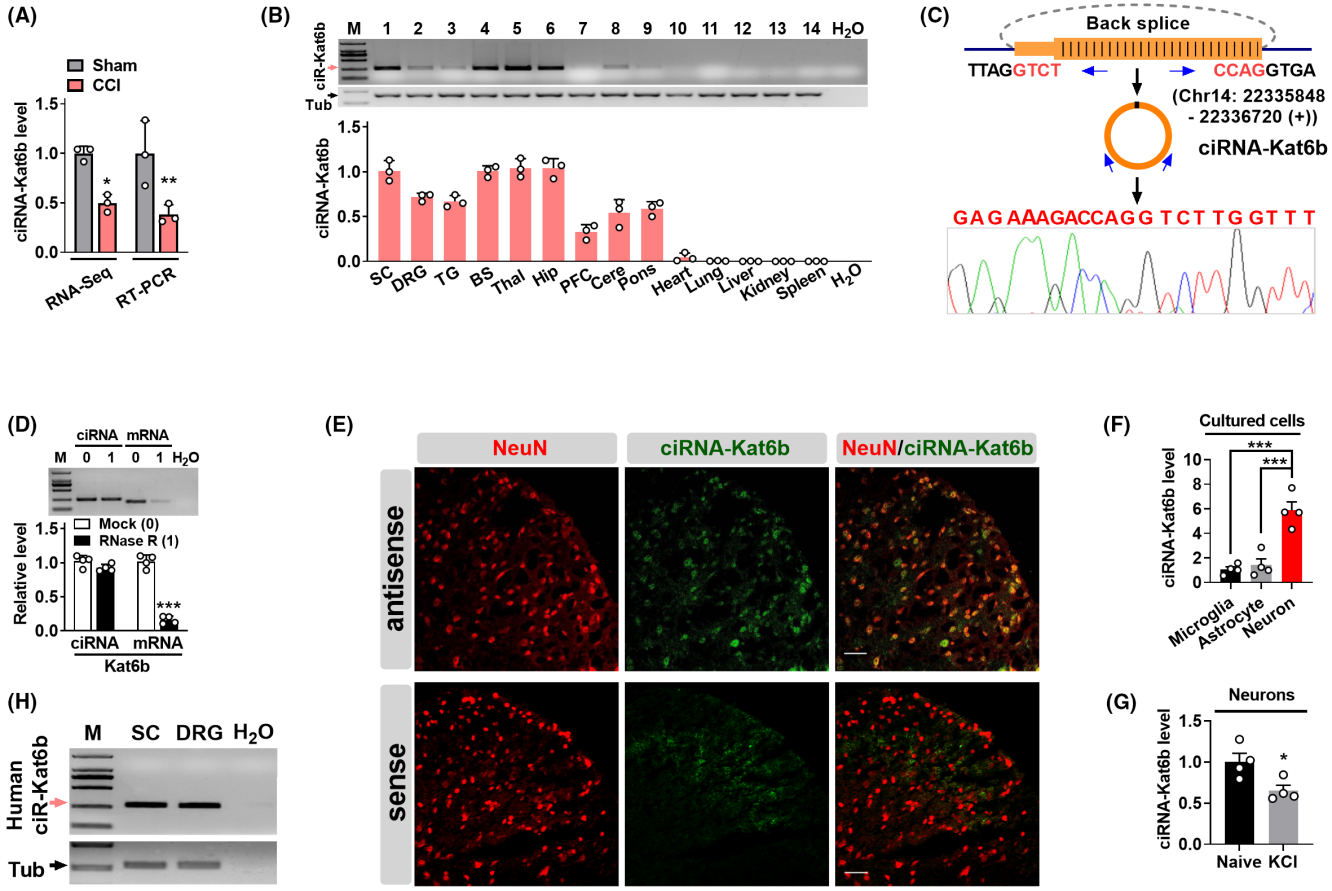
Identification of neuron‐specific circular RNA *Kat6b* (*ciRNA‐Kat6b*) in nervous system tissues. (A) Peripheral nerve injury led to the differential expression of *ciRNA‐Kat6b* in mouse spinal cord by the use of RNA‐Sequencing and RT‐qPCR. *n* = 3 mice/group. *p < 0.05, **p < 0.01 versus Sham groups by two‐tailed unpaired Student's *t*‐test. (B) *ciRNA‐Kat6b* was specifically expressed in nervous system tissues, such as the spinal cord, DRG (dorsal root ganglion), TG (trigeminal ganglion), BS (brain stem), thalamus, hippocampus, PFC (prefrontal cortex), cerebrum and pons, but not found in the non‐nervous system. *n* = 3 mice/group. (C) The *ciRNA‐Kat6b* location in chromosome and its circle identified by Sanger sequencing. The junction of ciRNA‐*Kat6b* was amplified by back‐to‐back primers and subjected to Sanger sequencing. (D) Resistance test of *ciRNA‐Kat6b* to RNase R digestion. Total RNAs were digested with RNase R and used for transcription and qRT‐PCR for *ciRNA‐Kat6b* and its parent *Kat6b* mRNA. *Kat6b* mRNA was detected as the RNase R‐sensitive control. *n* = 4 mice/group ***p < 0.001 versus the corresponding mock groups by two‐tailed unpaired Student's *t*‐test. (E) *ciRNA‐Kat6b* was mainly expressed in the spinal neuron. The detection was carried out by fluorescence in situ hybridization (FISH). NeuN, a neuron marker. Antisense indicates antisense probe; sense, sense probe, Scale Bar: 30 μm. (F) The relative level of circRNA‐*Kat6b* was analyzed by qRT‐PCR, respectively, in spinal neurons, astrocytes and microglial cells cultured in vitro. *n* = 4, ***p < 0.001 versus the corresponding groups by one‐way ANOVA followed by post hoc Tukey test. (G) circRNA‐*Kat6b* was decreased after treatment with KCl (50 mM) for 12 h in cultured spinal neurons. *n* = 4 per group. *p < 0.05 versus the related naive groups by two‐tailed paired Student's *t*‐test. (H) *CiRNA‐Kat6b* was detected in the human spinal cord and dorsal root ganglion using reverse transcription (RT)‐PCR with strand‐specific primers. To exclude genomic DNA contamination, the extracted RNA samples were pretreated with DNase I. �‐tubulin III was used as a control. *n* = 3 biological repeats/tissues. SC, spinal cord; M, DNA ladder marker. H_2_O was used for the negative control.

To explore the circRNA mechanisms underlying neuropathic pain, we carried out the deep sequencing of circRNAs from the ipsilateral spinal dorsal horn of mice subjected to chronic constriction injury (CCI) and Sham surgery. Among the differential expression ciRNAs (Table S1), *ciRNA‐Kat6b* was downregulated by 2.1 folds after peripheral nerve injury, this downregulation was further confirmed by qRT‐PCR (Figure 1A). Interestingly, although *ciRNA‐Kat6b* was not the most differential expression ciRNA, *ciRNA‐Kat6b* was specifically expressed in nervous system tissues including 9 mouse nervous tissues (including the spinal cord, dorsal ganglia root, thalamus, cerebellum, hippocampus, trigeminal ganglion, cortex, and brain stem), but undetected in 5 non‐nervous tissues (including heart, lung, liver, kidney, and spleen) (Figure 1B). Therefore, *ciRNA‐Kat6b* was chosen as the investigation target in this study. Further informatics analysis showed that *ciRNA‐Kat6b* was located on chromosome 14: 22,335,848–22,336,720 (+) and back spliced by exon 2 of *Kat6b* (or named *Myst4*) (Figure 1C). The distinct product of the expected size was amplified using outward‐facing primers, and its junction point was verified by Sanger sequencing (Figure 1C). To test the stability of *ciRNA‐Kat6b*, total spinal RNAs from naïve mice were treated with RNase R exonuclease, and the result showed that *ciRNA‐Kat6b* was resistant to RNase R digestion, whereas linear *Kat6b* mRNA was easily degraded, indicating that *ciRNA‐Kat6b* is the circular RNA in form (Figure 1D). To explore the expression distribution in cellular type and tissues, we carried out the test by fluorescence *in suit* hybridization (FISH). To exclude the effect of the linear *Kat6b*, we treated the spinal cord section with RNase R to digest the linear RNA. FISH result from the antisense probe showed that *ciRNA‐Kat6b* was mainly expressed in spinal cord neurons, but FISH results from the sense probe did not display a signal (Figure 1E). Given that fluorescent staining in the spinal slice only exhibits a single layer of cellular information in *ciRNA‐Kat6b* expression. To further evaluate the expression level of *circRNA‐Kat6b* in spinal neurons and non‐neurons, we respectively isolated the spinal neurons, astrocytes, and microglia from spinal cord tissues. After culture, we analyzed the *ciRNA‐Kat6b* level in three types of cells. The results showed that *ciRNA‐Kat6b* in neurons was 5.9‐fold as that in microglial cells and 4.3‐fold as that in astrocyte cells. These results together with FISH data supports the conclusion that *ciRNA‐Kat6b* was mainly expressed in spinal cord neurons (Figure 1F). Because the cultured neurons can be depolarized with high‐concentration KCl to mimic sensitized in vivo neurons by nociceptive response,^19^ we treated the cultured spinal neurons with 50 mM KCl for 12 h and found that *ciRNA‐Kat6b* was significantly decreased by this treatment (Figure 1G), supporting a consistent change trend of *ciRNA‐Kat6b* expression between the spinal neurons of CCI‐treated mice and KCl‐treated spinal neurons in vitro. Further analysis by the cirbase database showed that *ciRNA‐Kat6b* was conserved in mammal animals such as humans, rats, and dogs (Figure S1A). *CiRNA‐Kat6b* was also detected in the human spinal cord and DRG (Figure 1H). Collectively, we identified that *ciRNA‐Kat6b* is specifically expressed in the nervous system, and has high conservation between mice and humans.

### 3.2 Peripheral nerve injury decreased *ciRNA‐Kat6b* expression in the spinal cord

**FIGURE 2:**
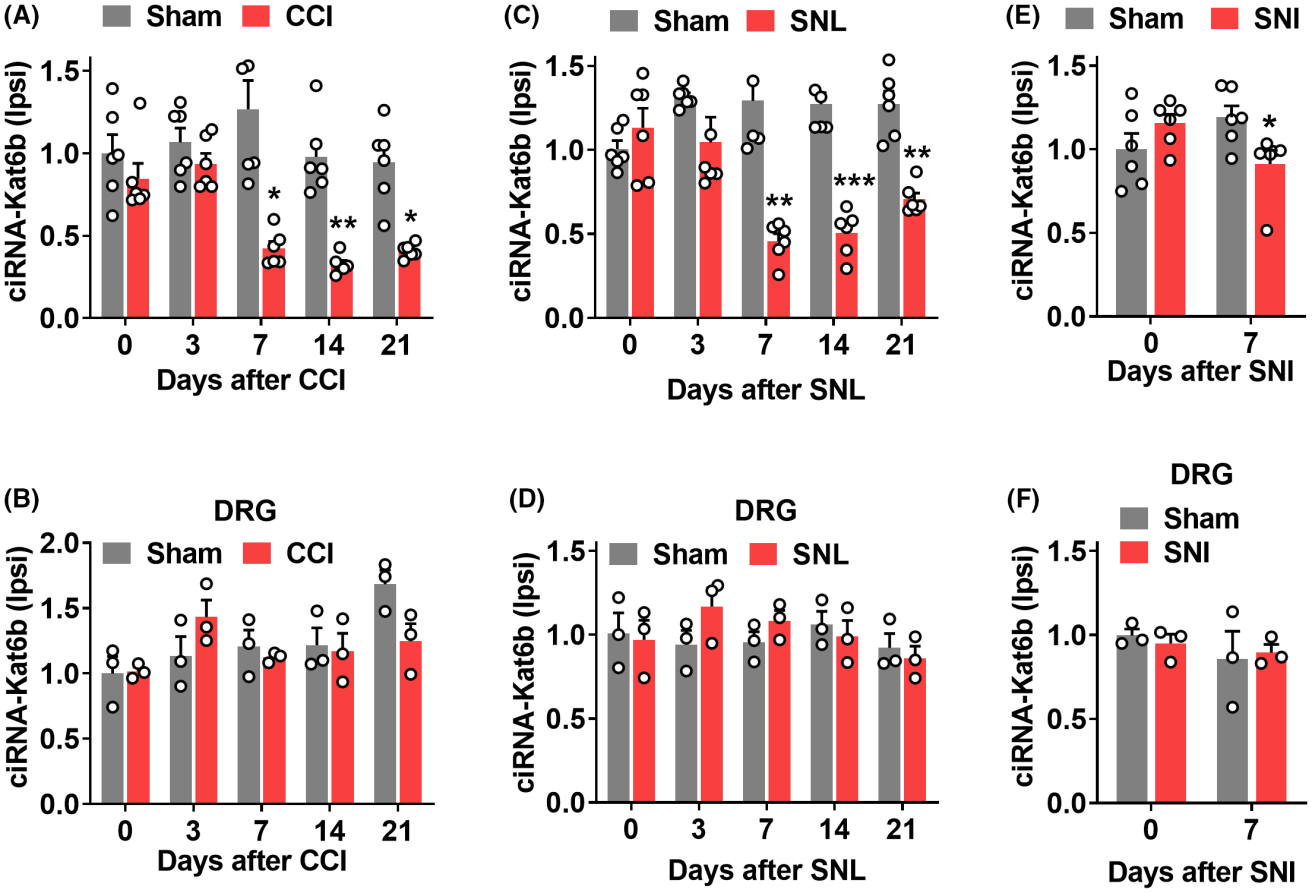
Downregulation of *ciRNA‐Kat6b* expression in the spinal cord after peripheral nerve injury. (A, B) Levels of *ciRNA‐Kat6b* in the ipsilateral dorsal spinal cord (A) and ipsilateral dorsal root ganglion (DRG) (B) after chronic constriction injury (CCI) or Sham surgery of unilateral sciatic nerve. *n* = 6 mice/time point/group. A total of 4 DRGs from two mice were pooled to be one sample. *P < 0.05, **P < 0.01 versus the corresponding Sham group by two‐way ANOVA followed by post hoc Tukey test. (C, D) Levels of *ciRNA‐Kat6b* in the ipsilateral dorsal spinal cord (C) and ipsilateral L4 DRG (D) after unilateral spinal nerve ligation (SNL) or Sham surgery. *n* = 6 mice/time point/group. **P < 0.01, ***P < 0.001 versus the corresponding Sham group by two‐way ANOVA followed by post hoc Tukey test. (E, F) Levels of *ciRNA‐Kat6b* in the ipsilateral dorsal spinal cord (E) and L4 DRGs (F) on day 7 after sciatic nerve injury surgery or Sham surgery. *n* = 6 mice/time point/group. *P < 0.05 versus the corresponding Sham group on day 0 by two‐way ANOVA followed by post hoc Tukey test. *n* = 6 mice/time point/group.

We next examined whether *ciRNA‐Kat6b* was altered in a time‐course‐dependent manner in the spinal cord after peripheral nerve injury. *CiRNA‐Kat6b* was significantly reduced in the ipsilateral (not contralateral; Figure S2A) dorsal spinal cord on days 7, 14, and 21 following chronic constriction injury (CCI) of the unilateral sciatic nerve, but not Sham surgery (Figure 2A). However, the change of *ciRNA‐Kat6b* was not detected in both the ipsilateral (Figure 2B) and the contralateral (Figure S2B) dorsal root ganglion (DRG) during the observed times from days 3 to 21 after surgery. The downregulation of *ciRNA‐Kat6b* was also observed in the ipsilateral dorsal spinal cord (Figure 2C), but not observed in the contralateral dorsal spinal cord (Figure S2C) and in both sides of DRG (Figure 2D and Figure S2D) after unilateral L4 spinal nerve ligation (SNL). Similar to CCI and SNL neuropathic pain model, unilateral sciatic nerve injury (SNI) led to a marked decrease of *ciRNA‐Kat6b* in the ipsilateral dorsal spinal cord (Figure 2E), however, did not change its expression in the contralateral spinal cord (Figure S2E), or in both ipsilateral and contralateral DRG (Figure 2F and Figure S2F) on day 7 after surgery. To further examine whether peripheral nociceptive stimulus changed the expression of *ciRNA‐Kat6b* in the dorsal spinal cord, we intra‐plantar‐injected the Complete Fraud's Adjuvant (CFA) to produce the chronic inflammation pain. The *ciRNA‐Kat6b* in the ipsilateral injection side was not altered from hour 2 to day 7 post‐CFA injection (Figure S2G). It thus appears that peripheral nerve injury but not peripheral inflammation down‐regulates *ciRNA‐Kat6b* in the dorsal spinal cord.

### 3.3 Rescuing downregulated spinal *ciRNA‐Kat6b* mitigates neuropathic pain

**FIGURE 3:**
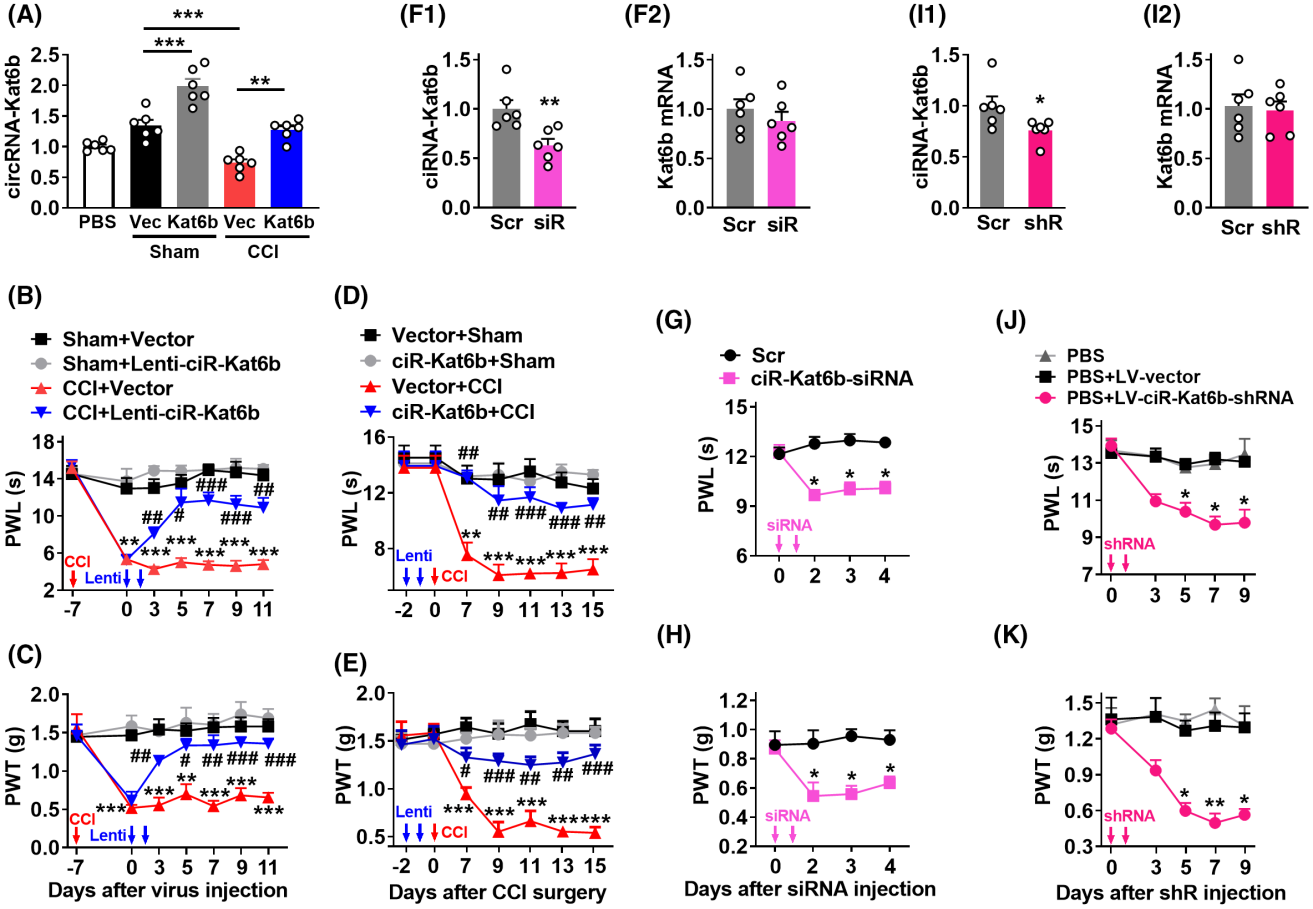
*CiRNA‐Kat6b* was involved in neuropathic pain development and maintenance. (A–E) Rescuing the nerve injury‐induced downregulation of *ciRNA‐Kat6b* attenuated neuropathic pain initiation and maintenance: (A) The *ciRNA‐Kat6b* expression in the dorsal spinal cord on day 5 after intrathecal injection of Lenti‐*Gfp* (GFP) or Lenti‐*ciRNA‐Kat6b* (*Kat6b*) into mice with 7 days CCI or Sham surgery. *n* = 6 mice/group. **P < 0.01, ***P < 0.001 versus the corresponding group by one‐way ANOVA followed by post hoc Tukey test. (B, C) Effect of intrathecal post‐injection of Lenti‐*ciRNA‐Kat6b* (Lenti‐ciR‐*Kat6b*) or Lenti‐*Gfp* (Vector) on the hind paw withdrawal latencies (PWL) to heat stimuli (B) and paw withdrawal threshold (PWT) to von Frey filaments stimuli (C) on the ipsilateral side at the different days after CCI surgery. *n* = 8 mice/group. **p < 0.01, ***p < 0.001 versus the Sham plus Vector group at the corresponding time points by two‐way ANOVA with repeated measures followed by post hoc Tukey test; ^#^ *p* < 0.05, ^##^ *p* < 0.01, ^###^ *p* < 0.001 versus the CCI plus Vector group at the corresponding time points by two‐way ANOVA with repeated measures followed by post hoc Tukey test. (D, E) Effect of intrathecal pre‐injection of Lenti‐*ciRNA‐Kat6b* (Lenti‐ciR‐*Kat6b*) or Lenti‐*Gfp* (Vector) on paw withdrawal latencies (PWL) to heat stimuli (D) and paw withdrawal threshold (PWT) to von Frey filaments stimuli (E) on the ipsilateral side at the different days after CCI or Sham surgery. *n* = 8 mice/group. **p < 0.01, ***p < 0.001 versus the Vector plus Sham group at the corresponding time points by two‐way ANOVA with repeated measures followed by post hoc Tukey test; ^#^ *p* < 0.05, ^##^ *p* < 0.01, ^###^ *p* < 0.001 versus the Vector plus CCI group at the corresponding time points by two‐way ANOVA with repeated measures followed by post hoc Tukey test. (F–K) Knockdown of spinal *ciRNA‐Kat6b* induced the generation of neuropathic pain‐like symptoms: F1–F2, Levels of *ciRNA‐Kat6b* (F1) or *Kat6b* (F2) in the dorsal spinal cord on day 3 after 2 consecutive days of intrathecal injection with *ciRNA‐Kat6b* siRNA (siRNA) or control scrambled siRNA (Scr) into the spinal cord. *n* = 6 mice/group. **p < 0.01 versus the scrambled siRNA group by two‐tailed unpaired Student's *t*‐test. (G–H) Effect of 2 consecutive days of intrathecal injection of *ciRNA‐Kat6b* siRNA or control scrambled siRNA (Scr) into spinal cord on paw withdrawal latencies (PWL) to heat stimuli (G) and paw withdrawal threshold (PWT) to von Frey filaments stimuli (H) at the different days after siRNA microinjection. *n* = 8 mice/group. *p < 0.05 versus the scrambled siRNA‐treated mice at the corresponding time points by two‐way ANOVA with repeated measures followed by post hoc Tukey test. I1‐I2, levels of *ciRNA‐Kat6b* (I1) or *Kat6b* (I2) in the dorsal spinal cord on day 6 after 2 consecutive days of intrathecal injection with LV‐*ciRNA‐Kat6b‐*shRNA (shRNA) or control scrambled LV‐Scr‐shRNA (Scr) into the spinal cord. *n* = 6 mice/group. *p < 0.05 versus the Scr group by two‐tailed unpaired Student's *t*‐test. (J, K) Effect of 2 consecutive days of intrathecal injection of LV‐*ciRNA‐Kat6b‐*shRNA or control scrambled LV‐Scr (LV‐vector) into spinal cord on paw withdrawal latencies (PWL) to heat stimuli (J) and paw withdrawal threshold (PWT) to von Frey filaments stimuli (K) at the different days after virus injection. *n* = 8 mice/group. *p < 0.05, **p < 0.01 versus the scrambled shRNA‐treated mice at the corresponding time points by two‐way ANOVA with repeated measures followed by post hoc Tukey test.

To examine whether spinal *ciRNA‐Kat6b* downregulation participates in the production of neuropathic pain, we rescued its downregulation through intrathecal of lentivirus (Lenti) expressing *ciRNA‐Kat6b* (Lenti‐*ciR‐Kat6b*) into spinal cord on day 7 after CCI or Sham surgery. The intrathecal injection of Lenti‐*ciR‐Kat6b* reversed the expression of *ciRNA‐Kat6b* on day 5 in the CCI group (Figure 3A). As described previously,^18^ CCI led to heat hyperalgesia (evidenced by decreased paw withdrawal latencies in response to heat) (Figure 3B) on the ipsilateral and mechanical allodynia (demonstrated by increased paw withdrawal threshold in response to *von Frey* filaments) (Figure 3C), but not contralateral (Figure S3A,B) sides of Lenti‐*Gfp* (used as a control)‐injected mice from day 7 to day 11 after CCI. These pain hypersensitivities were not observed in the CCI mice injected with Lenti‐*ciR‐Kat6b* during these periods (Figure 3B,C). Intrathecal injection of neither virus altered the basal paw responses to thermal and mechanical stimuli on the ipsilateral (Figure 3B,C) or contralateral (Figure S3A,B) side of the CCI mice and either side of the Sham mice. Spinal injection of Lenti‐*ciR‐Kat6b* on day 2 before CCI significantly attenuated thermal hyperalgesia and mechanical allodynia on days 7 and 15 post‐CCI on the ipsilateral side (Figure 3D,E). Expectedly, basal paw responses on the contralateral side (Figure S3C,D) and locomotor functions, measured by grasping, climbing, and righting reflex, were not affected (Table 1) in these mice.

**TABLE 1:** Mean changes in locomotor function.

Treatment groups	Locomotor function test		
	Placing	Grasping	Righting
PBS (5 μL)	8 (0)	8 (0)	8 (0)
Sham + Vector	8 (0)	8 (0)	8 (0)
Sham + Lenti‐ciR‐Katb6b	8 (0)	8 (0)	8 (0)
CCI + Vector	8 (0)	8 (0)	8 (0)
Scr	8 (0)	8 (0)	8 (0)
ciR‐*Kat6b*‐siRNA	8 (0)	8 (0)	8 (0)
PBS + LV‐vector	8 (0)	8 (0)	8 (0)
PBS + LV‐ciR‐*Kat6b*‐shRNA	8 (0)	8 (0)	8 (0)
Sham + Scr	8 (0)	8 (0)	8 (0)
Sham + miR‐26a Ih	8 (0)	8 (0)	8 (0)
CCI + Scr	8 (0)	8 (0)	8 (0)
CCI + miR‐26a Ih	8 (0)	8 (0)	8 (0)
Sham + LV‐Scr	8 (0)	8 (0)	8 (0)
Sham + miR‐26a shRNA	8 (0)	8 (0)	8 (0)
CCI + LV‐Scr	8 (0)	8 (0)	8 (0)
CCI + miR‐26a shRNA	8 (0)	8 (0)	8 (0)
miR‐26a mimics	8 (0)	8 (0)	8 (0)
Lenti‐Scr	8 (0)	8 (0)	8 (0)
Lenti‐miR‐26a	8 (0)	8 (0)	8 (0)
LV‐Scr + Scr	8 (0)	8 (0)	8 (0)
LV‐Scr + 26a‐Ih	8 (0)	8 (0)	8 (0)
LV‐ci‐K6b‐shR + Scr	8 (0)	8 (0)	8 (0)
LV‐ci‐K6b‐shR + 26a‐Ih	8 (0)	8 (0)	8 (0)
Sham + Gfp	8 (0)	8 (0)	8 (0)
Sham + *Kcnk1*	8 (0)	8 (0)	8 (0)
CCI + Gfp	8 (0)	8 (0)	8 (0)
CCI + *Kcnk1*	8 (0)	8 (0)	8 (0)
siR‐*Kcnk1*	8 (0)	8 (0)	8 (0)
Gfp + Scr	8 (0)	8 (0)	8 (0)
Gfp + 26a mimics	8 (0)	8 (0)	8 (0)
*Kcnk1* + Scr	8 (0)	8 (0)	8 (0)
*Kcnk1* + 26a mimics	8 (0)	8 (0)	8 (0)
26a + Gfp	8 (0)	8 (0)	8 (0)
Scr + *Kcnk1*	8 (0)	8 (0)	8 (0)
26a + *Kcnk1*	8 (0)	8 (0)	8 (0)

*Note*: Data are mean (SEM). *n* = 8/group; No significance; one‐way ANOVA (response time vs treated groups) followed by post hoc Tukey test.

### 3.4 Mimicking CCI‐induced spinal *ciRNA‐Kat6b* decrease leads to nociceptive hypersensitivity

We further examined whether spinal *ciRNA‐Kat6b* downregulation is sufficient for neuropathic pain induction. Tssso this end, *ciRNA‐Kat6b* siRNA (ciR‐*Kat6b*‐siRNA) was intrathecally injected in naïve adult mice. Scrambled siRNA was used as a control. *ciRNA‐Kat6b* siRNA that significantly knocked down *ciRNA‐Kat6b* in the dorsal spinal cord (Figure 3F1), but did not influence the expression of the linear *Kat6b* mRNA (Figure 3F2). The mice injected with *ciRNA‐Kat6b* siRNA, but not scrambled siRNA, exhibited significant decreases in paw withdrawal latencies to thermal and mechanical stimuli during the observed period from 2 to 4 days after injection (Figure 3G,H). Furthermore, we used lentivirus to endogenously produce shRNA (LV‐*ciR‐Kat6b*‐shRNA) to knock down *ciRNA‐Kat6b* in the spinal cord. *CiRNA‐Kat6b*, not the linear *Kat6b*, was decreased in the spinal cord on day 5 after intrathecal injection of LV‐*ciR‐Kat6b*‐shRNA (Figure 3I1,I2). Like the *ciRNA‐Kat6b* siRNA‐treated mice, the mice injected with LV‐*ciR‐Kat6b*‐shRNA, but not LV‐Scr, displayed both evoked thermal and mechanical sensitivities on 5, 7, and 9 after injection (Figure 3J,K). As expected, no changes in locomotor function (Table 1) were found in either siRNA or shRNA‐injected mice. Spinal ciRNA‐Kat6b downregulation likely caused neuropathic pain‐like symptoms.

### 3.5 *CiRNA‐Kat6b* acts to be a sponge absorbing *miRNA‐26a*

**FIGURE 4:**
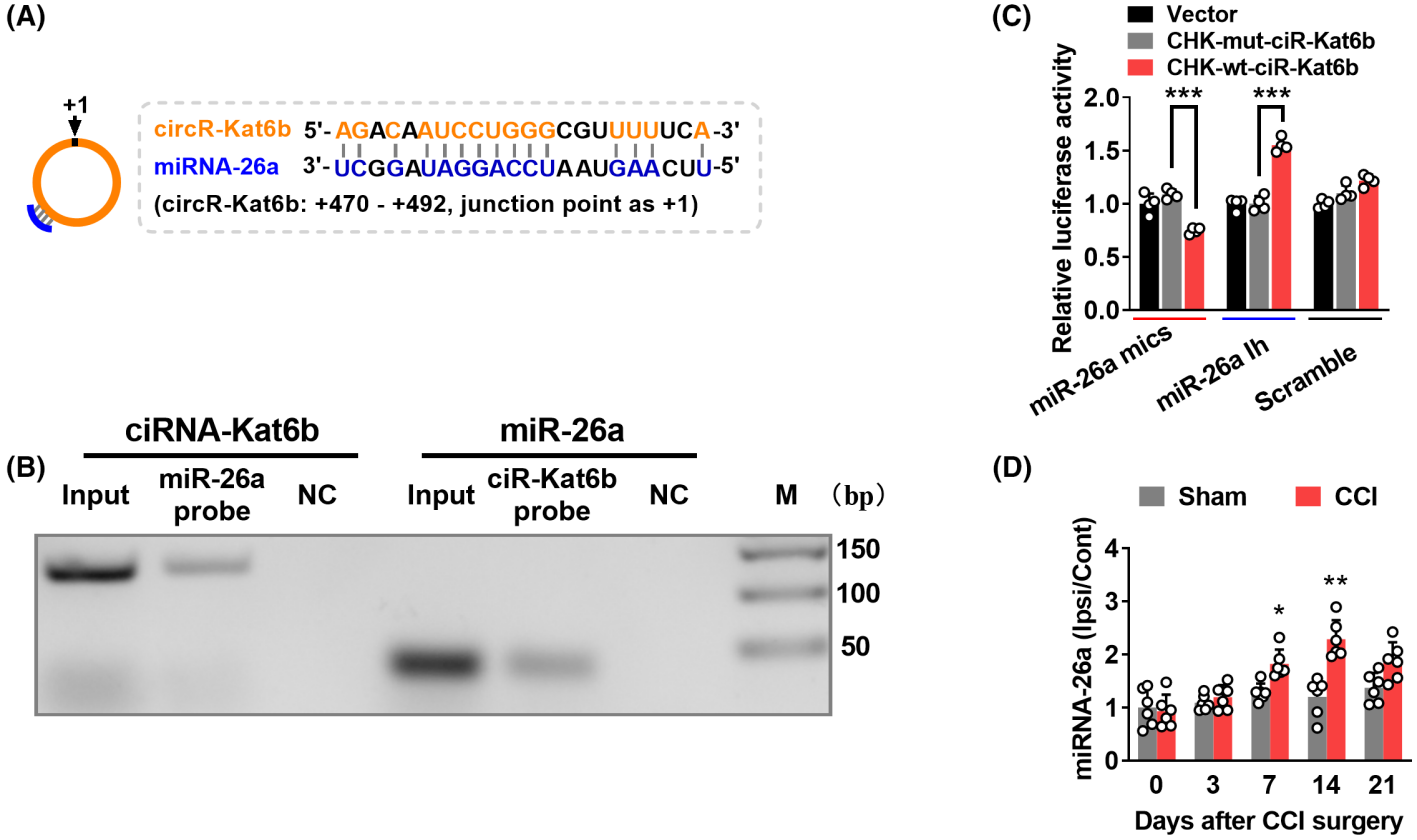
*CiRNA‐Kat6b* acts as competing endogenous RNAs to sponge *miRNA‐26a*. (A) Schematic presentation of *miRNA‐26a* binding to *ciRNA‐Kat6b*. (B) Identification of *miRNA‐26a* binding to *ciRNA‐Kat6b*: RT‐PCR detection for *ciRNA‐Kat6b* using the immunoprecipitated complex with *miRNA‐26a* (miR‐26a probe) or its mutated probe (NC) (left); or for *miRNA‐26a* using the immunoprecipitated complex with wild *ciRNA‐Kat6b* (ciR‐*Kat6b* probe) or its mutated probe (NC) (right). (C) The validation of *ciRNA‐Kat6b* negatively regulating *miRNA‐26a* by luciferase reporter assay in vitro. A fragment of *ciRNA‐Kat6b* containing the bound region by *miRNA‐26a* was inserted into pGL6 reporter vectors (CHK‐wt‐ciR‐*Kat6b*). A mutation was generated via altering the sequence bound by *miRNA‐26a* as indicated (CHK‐mut‐ciR‐*Kat6b*). The wild and mutation reporters were, respectively, co‐transfected into the HEK293T with *miRNA‐26a* mimics (miR‐26a mics) or inhibitor (miR‐26a Ih) or the scrambled. *n* = 4 per group. ***p < 0.001 versus CHK‐mut‐ciR‐*Kat6b*. Two‐way ANOVA (effect vs plasmid treated interaction) followed by post hoc Tukey test. (D) Levels of *miRNA‐26a* in the ipsilateral dorsal spinal cord and contralateral dorsal spinal cord after chronic constriction injury (CCI) or Sham surgery of unilateral sciatic nerve. *n* = 6 mice/time point/group. *p < 0.05, **p < 0.01 versus the corresponding Sham group by two‐way ANOVA followed by post hoc Tukey test.

How does spinal *ciRNA‐Kat6b* participate in neuropathic pain? We carried out a search using the *ciRNA‐Kat6b* sequence in the miRbase database, and found one possible 23 nt‐length bound regions (+470 to +492, junction point as +1) by *miRNA‐26a* in *ciRNA‐Kat6b* (Figure 4A). The RNA immunoprecipitation (RIP) assay revealed that a fragment of *ciRNA‐Kat6b* containing *miRNA‐26a*‐bound region could be amplificated from the complex immunoprecipitated with *miRNA‐26a* probe, but not its mutation control probe (Figure 4B), at the same time, *miRNA‐26a* was detectable from the immunoprecipitated complex with *circ‐Kat6b* probe (Figure 4B), indicating the binding ability of *miRNA‐26a* to *ciRNA‐Kat6b*. The luciferase assay revealed that co‐transfection of *miRNA‐26a* mimics (a synthesized small RNA with the same sequence mimicking its overexpression) and the wild pGL6 reporter with the binding fragment of *miRNA‐26a* to *ciRNA‐Kat6b* in luciferase promoter, significantly decreased the activity of the pGL6 reporter by 33% compared with the mutated pGL6 reporter (Figure 4C). Contrarily, the co‐transfection of *miRNA‐26a* inhibitor (Ih), a synthesized small RNA with its reverse complementary sequence used to knockdown *miRNA‐26a*, increased the activity of the wild reporter by 55%, compared with the mutated reporter (Figure 4C). Furthermore, we wondered whether neuropathic pain stimulus could regulate *miRNA‐26a* expression. We found that nerve injury upregulated the expression of spinal *miRNA‐26a* from day 3 to 21 after CCI surgery (Figure 4D). These data indicate that *ciRNA‐Kat6b* likely acts to be a sponge absorbing *miRNA‐26a* in spinal neurons.

### 3.6 *CiRNA‐Kat6b* regulates neuropathic pain by targeting *miRNA‐26a*

**FIGURE 5:**
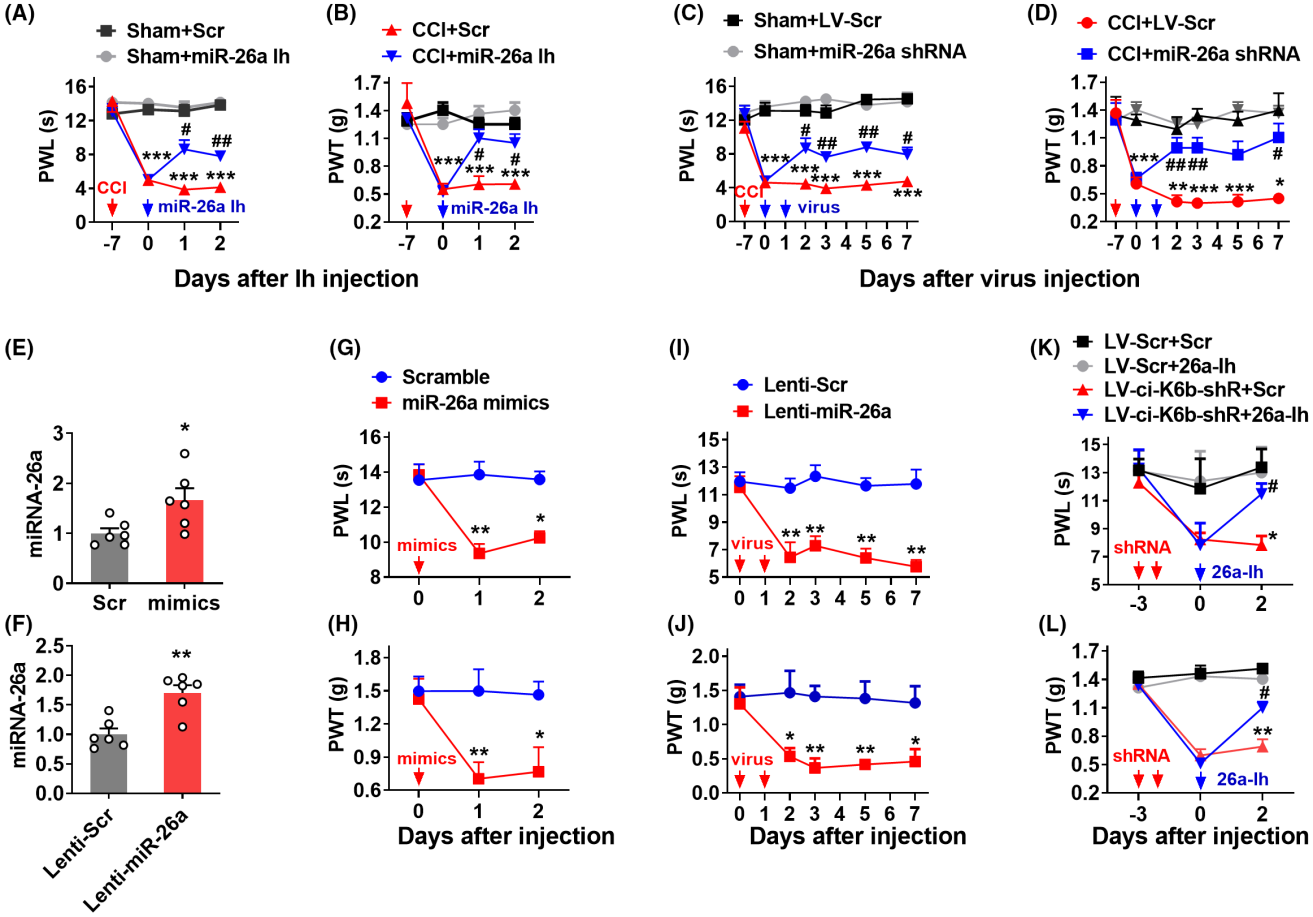
*MiRNA‐26a* upregulation contributes to neuropathic pain. (A, B) The intrathecal injections of *miRNA‐26a* inhibitor (miR‐26a Ih) but not its control scramble inhibitor (Scr) for 1 day alleviated the CCI‐induced thermal hyperalgesia (A) and mechanical allodynia (B). *n* = 8 mice/group. ***p < 0.001 versus the Sham plus Scr‐treated mice at the corresponding time points by two‐way ANOVA with repeated measures followed by post hoc Tukey test; ^#^ *p* < 0.05, ^##^ *p* < 0.01 versus the CCI plus Scr‐treated mice at the corresponding time points by two‐way ANOVA with repeated measures followed by post hoc Tukey test. Red arrow indicates CCI or Sham surgery. Blue arrows indicate miR‐26a Ih or Scr. (C, D) 2 consecutive days intrathecal injections of Lenti‐*miRNA‐26a* inhibitor (miR‐26a shRNA) but not control lentivirus‐mediated scramble inhibitor (LV‐Scr) attenuated thermal hyperalgesia (C) and mechanical allodynia (D) in CCI mice. *n* = 8 mice/group. *p < 0.05, ***p < 0.001 versus Sham plus LV‐Scr‐treated mice at the corresponding time points by two‐way ANOVA with repeated measures followed by post hoc Tukey test; ^#^ *p* < 0.05, ^##^ *p* < 0.01 versus CCI plus LV‐Scr‐treated mice at the corresponding time points by two‐way ANOVA with repeated measures followed by post hoc Tukey test. Red arrow indicates CCI or Sham surgery. Blue arrows indicate miR‐26a shRNA or LV‐Scr. (E) Levels of *miRNA‐26a* in the dorsal spinal cord on day 2 after intrathecal injection with *miRNA‐26a* mimics (mimics) or control scrambled miRNA (Scr) into spinal cord. *n* = 6 mice/group. *p < 0.05 versus the scrambled miRNA group by two‐tailed unpaired Student's *t*‐test. (F) Levels of *miRNA‐26a* in the dorsal spinal cord on day 5 after 2 consecutive days of intrathecal injection with Lenti‐*miRNA‐26a* (Lenti‐miR‐26a) or control scrambled virus (Lenti‐Scr) into the spinal cord. *n* = 6 mice/group. **p < 0.01 versus the scrambled virus group by two‐tailed unpaired Student's *t*‐test. (G, H) Intrathecal injection of *miRNA‐26a* mimics (miR‐26a mimics) or control scrambled miRNA (Scramble) into the spinal cord increased the hypersensitive response to heat stimuli (G) and to von Frey filaments stimuli (H) on the different days after mimics injection. *n* = 8 mice/group. *p < 0.05, **p < 0.01 versus the scrambled‐treated mice at the corresponding time points by two‐way ANOVA with repeated measures followed by post hoc Tukey test. The red arrow indicates mimics or Scr injection. (I, J) Two consecutive days of intrathecal injection of Lenti‐*miRNA‐26a* (Lenti‐miR‐26a) or control scrambled virus (Lenti‐Scr) into the spinal cord increased the hypersensitivity to heat stimuli (I) and to von Frey filaments stimuli (J) at the different days after lentivirus injection. *n* = 8 mice/group. *p < 0.05, **p < 0.01 versus the Lenti‐Scr‐treated mice at the corresponding time points by two‐way ANOVA with repeated measures followed by post hoc Tukey test. The red arrow indicates Lenti‐miR‐26a or Lenti‐Scr. (K, L) Two consecutive days intrathecal injection of *miRNA‐26a* inhibitor (26a‐Ih) on day 3 after intrathecal injection of Lenti‐*ciRNA‐Kat6b*‐shRNA (LV‐ci‐K6b‐shR) or its scrambled virus control (LV‐Scr) in naïve mice inhibited the production of pain hypersensitivity to heat stimuli (K) and to von Frey filaments stimuli (L). *n* = 8 mice/group. *p < 0.05, **p < 0.01 versus the LV‐Scr plus the scrambled inhibitor (Scr) group; ^#^ *p* < 0.05 versus the LV‐ci‐K6b‐shR plus Scr group by two‐way ANOVA with repeated measures followed by post hoc Tukey test. The red arrow indicates LV‐ci‐K6b‐shR or LV‐Scr injection. Blue arrows indicate 26a‐Ih or its control.

To explore whether *miRNA‐26a* could mediate the neuropathic pain induced by *ciRNA‐Kat6b* decrease, we measured the paw withdrawal responses to heat and mechanical stimulus after manipulating *miRNA‐26a* expression. Firstly, *miRNA‐26a* inhibitor (Ih) was intrathecally injected into the mice subjected to 7‐day CCI surgery. Scrambled small RNA (Scr) was used as a control. The mice injected with *miRNA‐26a*‐Ih, but not Scr, showed the attenuated response in paw withdrawal latencies to thermal and mechanical stimuli on day 1 after injection (Figure 5A,B). Similarly, the injected mice with LV‐*miRNA*‐26a shRNA (miR‐26a shRNA), producing *miRNA‐26a* inhibitor, but not its control LV‐Scr, displayed the alleviated pain hypersensitivity to thermal and mechanical stimulus (Figure 5C,D) from days 2 to 7 after 2 consecutive days of intrathecal injection. However, locomotor impairment, measured by reflex tests including placing, grasping, and righting as previously described^9^ was not observed after synthesized or lentivirus‐producing *miRNA‐26a* inhibitor (Table 1), suggesting that the decreased *miRNA‐26a* in the spinal cord is helpful to the alleviated of neuropathic pain.

Conversely, the other two modulation tools including *miRNA‐26a* mimics and Lenti‐*miRNA‐26* were employed to overexpress the *miRNA‐26a* in the spinal cord. In vivo test showed that *miRNA‐26a* mimics or Lenti‐*miRNA‐26* increased the content of *miRNA‐26a* by 66% or 70% respectively, indicating both its mimics and Lentivirus tools are effective in work (Figure 5E,F). Furthermore, intrathecal injection of *miRNA‐26a* mimics, but not scrambled control, for 1 day produced a nociception sensitivity response as evidenced by the hypersensitivity for thermal and mechanical stimulus in naïve mice (Figure 5G,H). A similar reduction in pain threshold was observed after *miRNA‐26a* overexpression with Lenti‐*miRNA‐26a*, but not negative control in naïve mice (Figure 5I,J), indicating the spinal *miRNA‐26a* overexpression leads to pain‐like symptoms. Whereas, the reflex tests confirmed no impairment of locomotor function after upregulating *miRNA‐26a* with mimics or lentivirus (Table 1). To further investigate whether the *ciRNA‐Kat6b*‐induced pain hypersensitivity was mediated through *miRNA‐26a*, we pre‐treated with LV‐*ciRNA‐Kat6b* (LV‐ci‐K6b‐shR) on day 3 before *miRNA‐26a* inhibitor (26a‐Ih) in naïve mice. We found intrathecal injection of *miRNA‐26a* inhibitor, but not its scrambled control, markedly relieved the pain hypersensitivity induced by the knockdown of *ciRNA‐Kat6b* (Figure 5K,L). Our data strongly suggest that spinal *ciRNA‐Kat6b* regulates neuropathic pain via the mediation of spinal *miRNA‐26a*.

### 3.7 Upregulation of *miRNA‐26a* decreases the expression of *Kcnk1* in spinal cord

**FIGURE 6:**
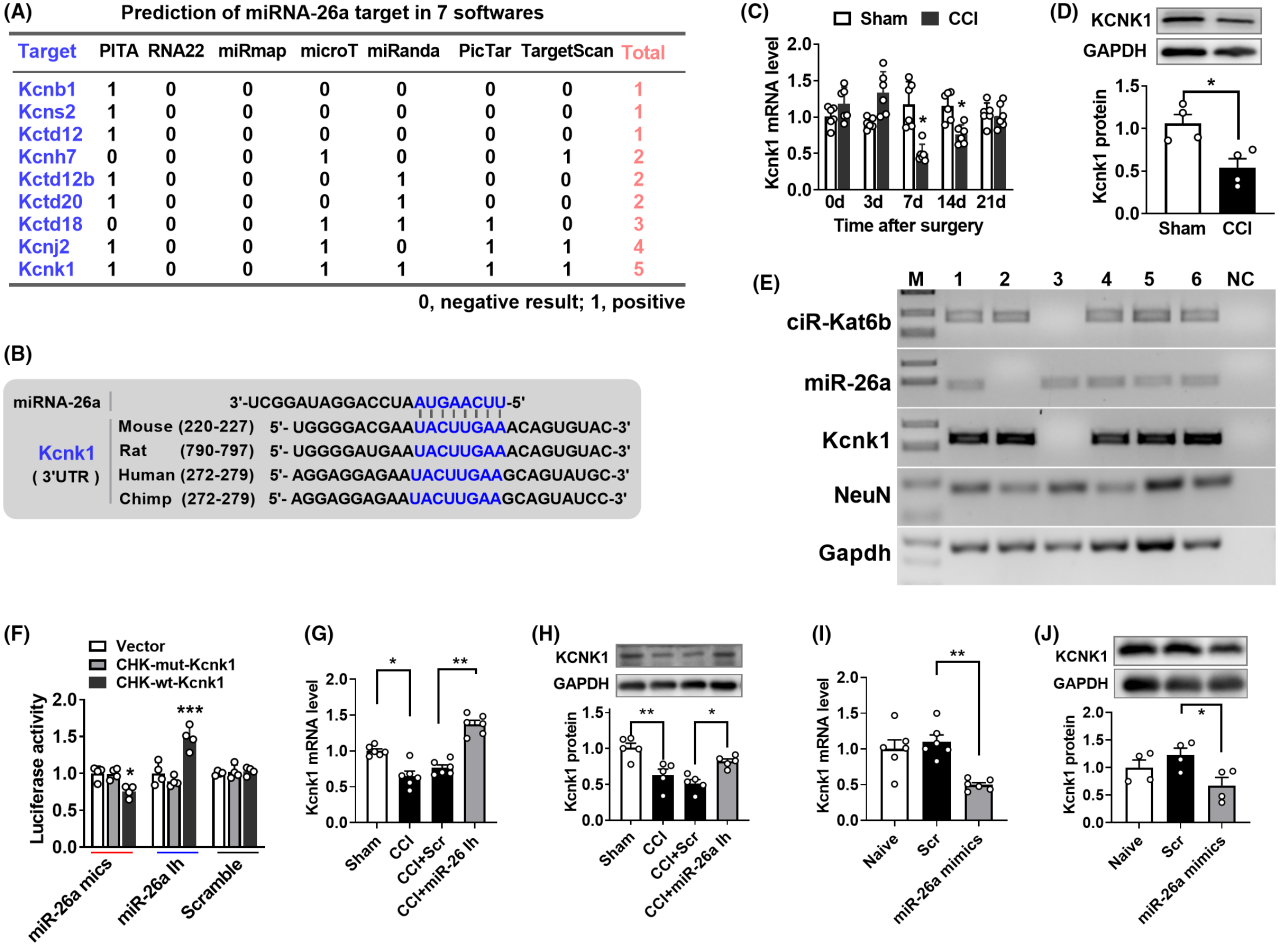
Upregulated *miRNA‐26a* was required for CCI‐induced decreases in *Kcnk1* expression in nerve‐injured spinal cord. (A) The sequence analysis of *miRNA‐26a* matching to 3′UTR of nine potassium ion channel mRNA by seven prediction programs including PITA, RNA22, miRmap, microT, miRanda, PicTar, and TargetScan. (B) The analysis of species conservation of *miRNA‐26a* targeting 3′UTR of *Kcnk1* mRNA among four mammal animals. (C) Peripheral nerve injury downregulated the expression of *Kcnk1* mRNA in a time‐dependent manner in the ipsilateral dorsal spinal cord after chronic constriction injury (CCI). *n* = 6 mice/time point/group. *p < 0.05, versus the corresponding Sham group by two‐way ANOVA followed by post hoc Tukey test. (D) The KCNK1 protein decreased in the ipsilateral dorsal spinal cord after CCI. *n* = 4 mice/group. *p < 0.05 versus the Sham group. (E) Co‐expression analysis of *ciRNA‐Kat6b*, *miRNA‐26a*, and *Kcnk1* in the single spinal neuron by single cell‐RT‐PCR. The number presented different samples. H_2_O was used for the negative control. *Gapdh* was used as the reference gene. (M) DNA ladder marker. (F) Verification of *Kcnk1* as a target gene of *miRNA‐26a* by luciferase reporter assay after the co‐transfection of luciferase reporter (CHK‐wt‐*Kcnk1*) with *miRNA‐26a* mimics (miR‐26a mics) or its scrambled control and *miRNA‐26a* inhibitor (miR‐26a Ih) or its scrambled control in HEK293T cells. A mutation (CHK‐mut‐*Kcnk1*) was generated in the *miRNA‐26a* matched seed sequence of *Kcnk1* mRNA‐3′UTR. *n* = 4. *p < 0.05, ***p < 0.001 versus the corresponding group by two‐way ANOVA followed by post hoc Tukey test. (G, H) The expression of *Kcnk1* mRNA (G) and protein (H) on day 2 after intrathecal injection of *miRNA‐26a* inhibitor (miR‐26a Ih) or its scrambled control (Scr) into the mice with 7‐day CCI or Sham surgery mice. *n* = 6 (G) or *n* = 5 (H). *p < 0.05, **p < 0.01 versus the corresponding group by one‐way ANOVA followed by post hoc Tukey test. (I, J) The expression of *Kcnk1* mRNA (I) and protein (J) on day 2 after intrathecal injection of *miRNA‐26a* mimics (miR‐26a‐mimics) or its scrambled control (Scr) into naïve mice. *n* = 6 (I) or *n* = 4 (J). *p < 0.05, **p < 0.01 versus the scrambled control group by one‐way ANOVA with post hoc Tukey test.

To explore how spinal *miRNA‐26a* increase is involved in neuropathic pain, we predicted its downstream targets by combining seven independent programs (Figure 6A). As the potassium channels paly a critical role in the regulation of neuronal excitability under chronic pain conditions,^13^, ^20^ we focused on the potassium channels among the predicted targets by *miRNA‐26a*. We found 9 potassium channel genes might become *miRNA‐26a*'s potential downstream targets (Figure 6A). Among them, *Kcnk1* (one member of the two‐pore domain background potassium (K2P) channel family) is likely the target of *miRNA‐26a* due to its highest frequency appearance by 5 prediction programs (Figure 6A). The 3′UTR of mouse *Kcnk1* mRNA constitutes a conserved *miR‐26a* binding sequence of “UACUUGAA” (220‐227 bp, the first base of 3′UTRas +1), and this region displayed a high specific conversation among 4 mammal specifics including mouse, rat, human, and chimp (Figure 6B). KCNK1 is a critical player in the modulation of the neuropathic pain through controlling K^+^ current in peripheral sensory neurons.^17^ Therefore, *Kcnk1* was chosen as a potential target of *miRNA‐26a* in this study. Our findings showed that peripheral nerve injury also decreased *Kcnk1* mRNA expression in the ipsilateral dorsal spinal cord from the day 3 to day 21 after surgery (Figure 6C). The KCNK1 protein in the ipsilateral spinal dorsal cord was also decreased on day 7 after CCI surgery (Figure 6D). Additionally, single‐cell PCR showed that *Kcnk1*, *miRNA‐26a*, and *ciRNA‐Kat6b* were co‐expressed in the spinal neuron (Figure 6E). Thus, we wondered whether *miRNA‐26a* could regulate the expression of KCNK1 in the dorsal spinal cord. Further studies between the in vitro and in vivo experiments were carried out to verify this point. In vitro, we cloned the 3′UTR of mouse *Kcnk1* mRNA containing the region bound by *miRNA‐26a* into the firefly luciferase reporter (CHK‐wt‐kcnk1). As expected, the luciferase assay revealed that co‐transfection of wild‐type reporter (CHK‐wt‐kcnk1), but not control mutated reporter (CHK‐mut‐kcnk1), with *miRNA‐26a* mimics significantly decreased the activity of the luciferase in HEK293T cells (Figure 6F). Contrarily, the co‐transfection of wild‐type but not mutated reporter with *miRNA‐26a* inhibitor increased the activity of the luciferase (Figure 6F). Furthermore, knocking down the CCI‐induced *miRNA‐26a* increase in the spinal cord through intrathecal injection of *miRNA‐26a* inhibitor (but not Scr) reversed the CCI‐induced decreases in *Kcnk1* in the ipsilateral dorsal spinal cord (Figure 6G,H). Additionally, spinal upregulation of *miRNA‐26a* through intrathecal injection of *miRNA‐26a* mimics (but not Scr) reduced the levels of dorsal spinal cord *Kcnk1* mRNA (Figure 6I) and protein (Figure 6J). These in vitro and in vivo data strongly support the idea that nerve injury‐induced KCNK1 downregulation is due, at least in part, to increased *miRNA‐26a* expression in the spinal cord of mice with nerve injury.

### 3.8 The decrease of *Kcnk1* is required for the pain hypersensitivity induced by *miRNA‐26a*

**FIGURE 7:**
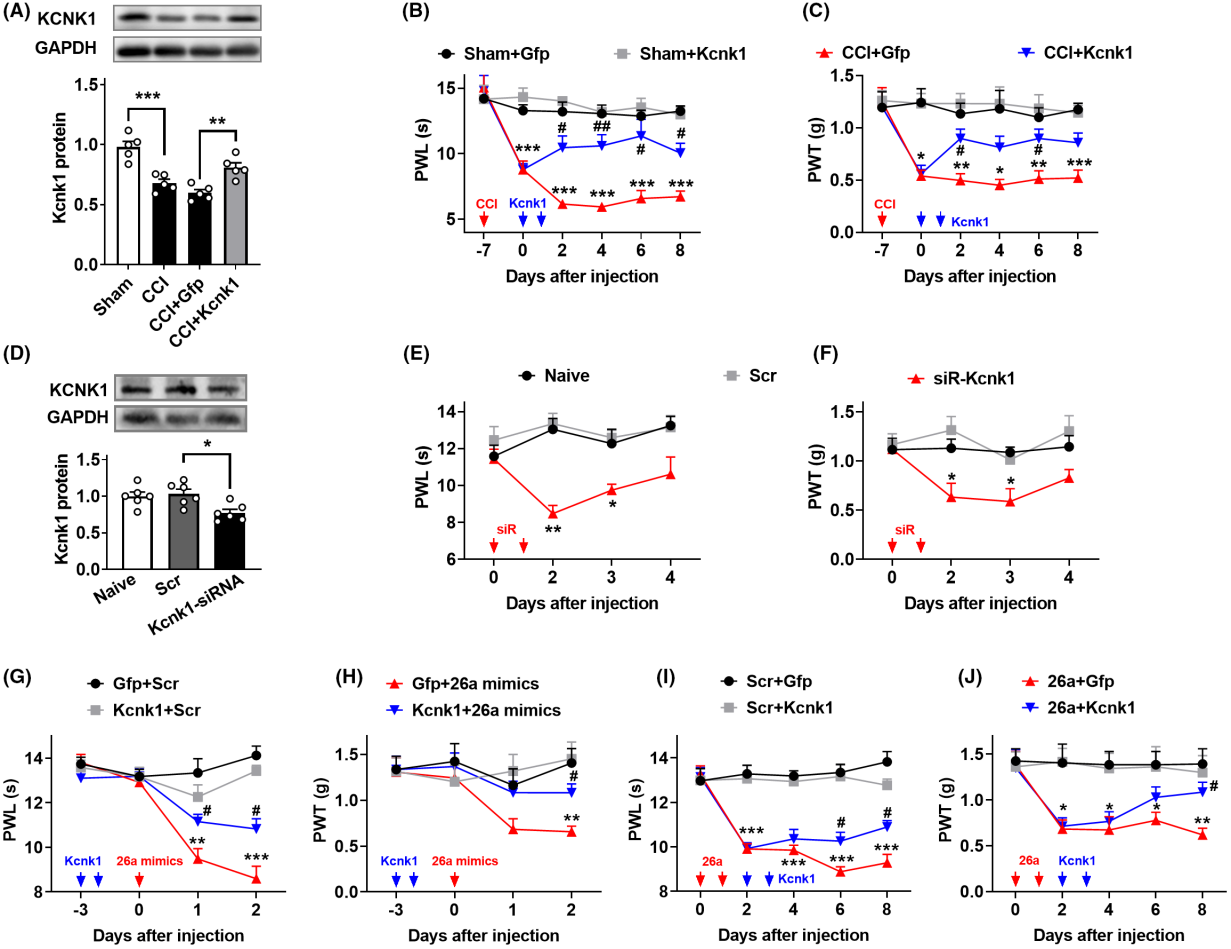
*MiRNA‐26a* regulates neuropathic pain via the mediation of KCNK1. (A) Levels of KCNK1 in the dorsal spinal cord on day 5 after 2 consecutive days of intrathecal injection with Lenti‐*Kcnk1* (*Kcnk1*) or its control Lenti‐*Gfp* (Gfp) day 7 post‐CCI or Sham surgery. *n* = 5 mice/group. **p < 0.01, ***p < 0.001 versus the corresponding groups by one‐way ANOVA with post hoc Tukey test. (B, C) The intrathecal injections of Lenti‐*Kcnk1* (*Kcnk1*) but not its control Lenti‐*Gfp* (Gfp) for 2 consecutive days attenuated the sensitivity response to heat stimuli (B) and to von Frey filaments stimuli (C). *n* = 8 mice/group. *p < 0.05, **p < 0.01, ***p < 0.01 versus the Sham plus Lenti‐*Gfp*‐treated mice at the corresponding time points by two‐way ANOVA with repeated measures followed by post hoc Tukey test; ^#^ *p* < 0.05, ^##^ *p* < 0.01 versus the CCI plus Lenti‐*Gfp*‐treated mice at the corresponding time points by two‐way ANOVA with repeated measures followed by post hoc Tukey test. The red arrow indicates CCI or Sham surgery. Blue arrows indicate Lenti‐*Kcnk1* or Lenti‐*Gfp* injection. (D) Levels of KCNK1 in the spinal dorsal cord on day 2 after 2 consecutive days of intrathecal injection of *Kcnk1* siRNA or the scrambled siRNA (Scr) in naïve mice. *n* = 4 mice/group. *p < 0.05 versus the scrambled siRNA group by one‐way ANOVA with post hoc Tukey test. (E, F) The intrathecal injections of *Kcnk1* siRNA (siR‐*Kcnk1*) but not the scrambled siRNA (Scr) for 2 consecutive days induced the neuropathic pain‐like symptom evidenced by heat hyperalgesia (E) and mechanical allodynia (F) in naïve mice. *n* = 8 mice/group. *p < 0.05, **p < 0.01 versus the scrambled siRNA‐treated mice at the corresponding time points by two‐way ANOVA with repeated measures followed by post hoc Tukey test. The red arrow indicates siRNA injection. (G, H) Intrathecal pre‐injection of Lenti‐*Kcnk1* (*Kcnk1*) for two consecutive days prevented the thermal hyperalgesia (G) and mechanical allodynia (H) induced by *miRNA‐26a* mimics (26a mimics) during the development period. *n* = 8 mice/group. **p < 0.01, ***p < 0.001 versus the scrambled Lenti‐*Gfp* plus miRNA scramble‐treated mice at the corresponding time points by two‐way ANOVA with repeated measures followed by post hoc Tukey test; ^#^ *p* < 0.05 versus the Lenti‐*Gfp* plus *miRNA‐26a* mimics‐treated mice at the corresponding time points by two‐way ANOVA with repeated measures followed by post hoc Tukey test. The red arrow indicates *miRNA‐26a* mimics or Scr injection. The blue arrow indicates Lenti‐*Kcnk1* or Lenti‐*Gfp* injection. (I, J) Intrathecal post‐injection of Lenti‐*Kcnk1* (*Kcnk1*) for 2 consecutive days inhibited the pain hypersensitivity response to thermal and mechanical stimulus induced by Lenti‐*miRNA‐26a* (26a) or Lenti‐Scr (Scr) during the maintenance period. *n* = 8 mice/group. *p < 0.05, **p < 0.01, ***p < 0.001 versus the scrambled Lenti‐Scr plus Lenti‐*Gfp*‐treated mice at the corresponding time points by two‐way ANOVA with repeated measures followed by post hoc Tukey test; ^#^ *p* < 0.05 versus the scrambled Lenti‐miRNA‐*26a* plus Lenti‐*Gfp*‐treated mice at the corresponding time points by two‐way ANOVA with repeated measures followed by post hoc Tukey test. The red arrow indicates Lenti‐*miRNA‐26a* or Lenti‐Scr injection. The blue arrow indicates Lenti‐*Kcnk1* or Lenti‐*Gfp* injection.

To further investigate whether spinal *Kcnk1* is involved in the development of neuropathic pain, we used the lentivirus to overexpress *Kcnk1* in the spinal cord of neuropathic pain mice. We found *Kcnk1* was increased on day 5 after the intrathecal injection of Lenti‐*Kcnk1* but its control Lenti‐*Gfp* (Figure 7A), and its upregulation rescued the decrease of spinal *Kcnk1* in the nerve injury mice (Figure 7A). Furthermore, rescuing the CCI‐induced downregulation in spinal *Kcnk1* through Lenti‐*Kcnk1*, but not Lenti‐*Gfp*, attenuated the induction of CCI‐induced thermal and mechanical pain hypersensitivities (Figure 7B,C) with no locomotor impairment (Table 1). Additionally, spinal knockdown of *Kcnk1* through intrathecal injection of *Kcnk1* siRNA, not scrambled siRNA, reduced the levels of *Kcnk1* in the spinal cord of naïve mice (Figure 7D) and augmented the responses to thermal and mechanical stimuli after siRNA injection (Figure 7E,F), indicating the pain‐like behavior genesis. Expectedly, the locomotor injury was not affected by siRNA or scramble control (Table 1).

Finally, to test whether *miRNA‐26a* modulates pain hypersensitivity via the mediation of *Kcnk1*, we increased the expression of *Kcnk1* post or before *miRNA‐26a* in the spinal cord of naïve mice. We found that the intrathecal pre‐injection of Lenti‐*Kcnk1* on day 3 before *miRNA‐26a* mimics injection prevented the *miRNA‐26a* increase‐induced nociception sensitivity to thermal and mechanical stimulus in naïve mice (Figure 7G,H). Similarly, the intrathecal injection of Lenti‐*Kcnk1* post‐injection of Lenti‐*miRNA‐26a* abolished the *miRNA‐26a* upregulation‐induced pain sensitivity in naïve mice (Figure 7I,J). These data suggested that *Kcnk1* mediates the pain hypersensitivity induced by the *miRNA‐26a* increase.

## 4 DISCUSSION

**FIGURE 8:**
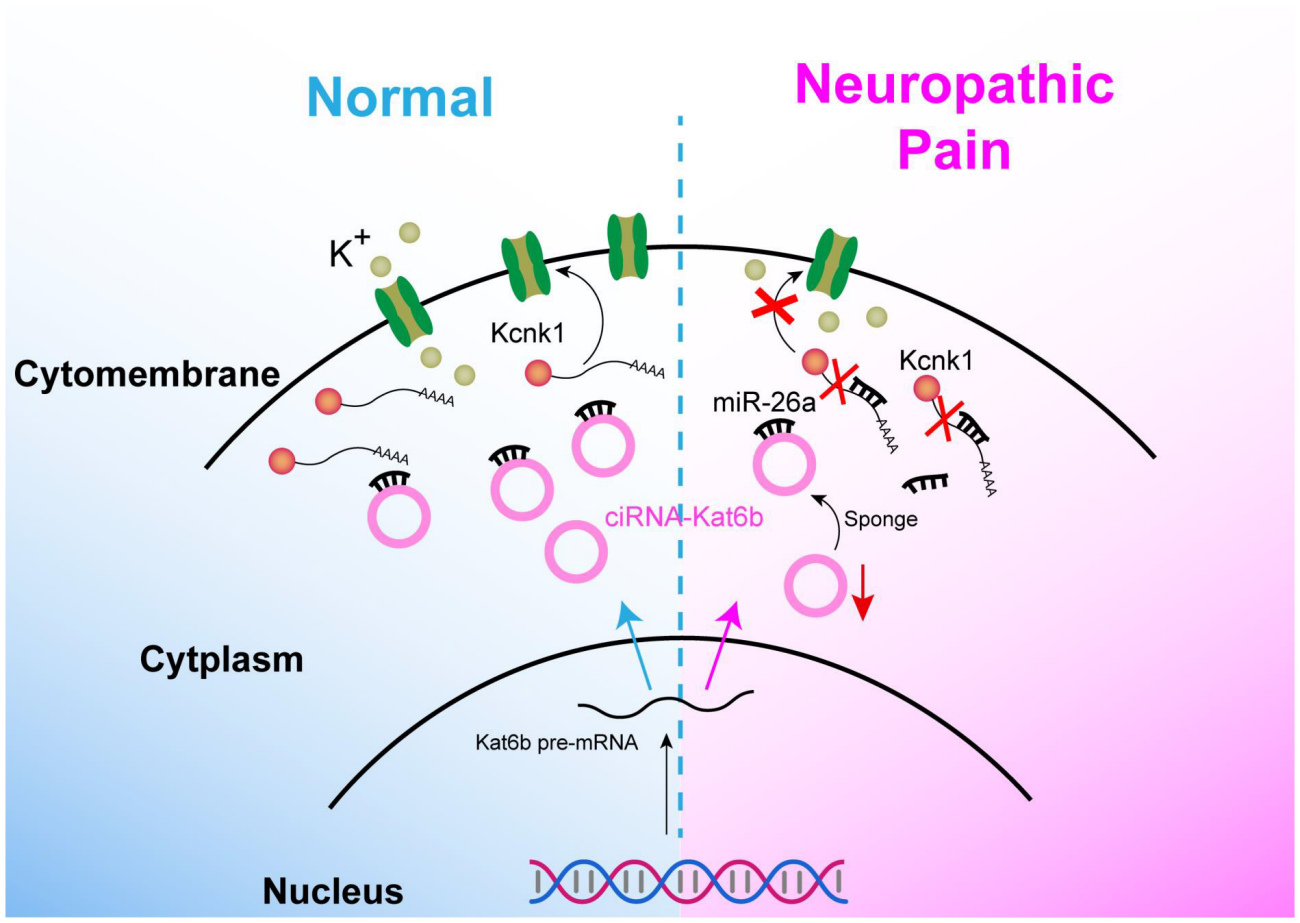
Proposed mechanism by which ciRNA‐*Kat6b* contributes to neuropathic pain.

circRNAs have been emerging to be a novel regulatory function mechanism in gene expression and attract widespread attention for their key roles in myriad biological processes and human diseases.^21^, ^22^ Bioinformatic analyses from human brain tissues identified a large number of neuronal‐specific circRNAs, 80% of mouse brain circRNAs are also detected in the human brain. The expression levels of these circRNAs are often changed during neuronal differentiation and in nervous system‐related diseases,^23^ but how they are causally linked to neuronal‐specific physiological and pathological functions remains elusive. In this study, we identified a nervous system‐specific circRNA—*ciRNA‐Kat6b* and reported its downregulation in the spinal cord following peripheral nerve injury. This downregulation was required for the induction and maintenance of neuropathic pain through positive regulation of *miRNA‐26a*‐triggered *Kcnk1* in the spinal cord. *CiRNA‐Kat6b* likely is a critical player in the mechanisms of nerve injury‐induced pain hypersensitivity (Figure 8).

*CiRNA‐Kat6b* downregulation is required for the *miRNA‐26a* increase in the spinal cord of neuropathic pain mice. Circular non‐coding RNAs exert important biological functions through their interaction with other RNA, DNA, and protein in gene expression.^24^ We demonstrated *ciRNA‐Kat6b* acts as an *miRNA‐26a* sponge to regulate the *miRNA‐26a* level and triggered *Kcnk1* mRNA content in the spinal cord. The lentivirus‐mediating upregulation of *ciRNA‐Kat6b* blocked the increased level of *miRNA‐26a* in the spinal cord of CCI mice, resulting in the attenuation of pain hypersensitivity. The downregulation of *ciRNA‐Kat6b* with siRNA or shRNA led to the increase of *miRNA‐26a* in the spinal cord of naïve mice, causing the neuropathic pain‐like behavior genesis. MicroRNAs have been demonstrated to be closely linked to a variety of pain types, ranging from primary nerve tissue such as DRG^25^ to the central nervous system such as spinal cord^26^ and pain‐related brain regions.^27^ *MiRNA‐26a* is involved in several diseases as neonatal sepsis,^28^ stroke,^29^ coronary heart disease (CHD),^30^ and cancer.^31^ But it remains unknown what the expression pattern of *miRNA‐26a* is and how it is regulated in nervous system diseases. Here, we found *miRNA‐26a* is likely a key regulator in the initiation and maintenance of neuropathic pain. The inhibition of *miRNA‐26a* expression via the intrathecal injection of its exo‐ or endogenous reverse complementary small RNA rescued the reduction of pain threshold in CCI mice. Furthermore, the overexpression of *miRNA‐26a* prevented the production of nociception sensitivity induced by *ciRNA‐Kat6b* downregulation. *CiRNA‐Kat6b* appears to be a critical player in neuropathic pain via regulating *miRNA‐26a* content in the spinal cord. However, (1) it is still unclear how *ciRNA‐Kat6b* regulates the expression of *miRNA‐26a*; (2) the other potential mechanisms such as binding protein, DNA, or long noncoding RNA by which *ciRNA‐Kat6b* participates in neuropathic pain cannot be excluded; (3) how the decreased spinal *ciRNA‐Kat6b* is regulated under the condition of neuropathic pain. These issues in neuropathic pain remain to be further investigated in the future.

Downregulated spinal *ciRNA‐Kat6b* contributes to increasing the level of spinal *miRNA‐26a* and induces nociceptive hypersensitivity. The present study demonstrated that suppressed *miRNA‐26a* expression through intrathecal injection of *miRNA‐26a* inhibitor in CCI mice reversed the decreases in *Kcnk1* in the injured spinal cord, thereby abolishing neuropathic pain development and maintenance. Mimicking nerve injury‐induced spinal *miRNA‐26a* upregulation through intrathecal injection of *miRNA‐26a* mimics in naïve mice decreased the expression of *Kcnk1* and augmented the animals' response to noxious stimuli. *Kcnk1* is an endogenous initiator of neuropathic pain.^17^ *Kcnk1* (K2P 1.1) is the first found member in the family of the two‐pore domain potassium (K2P) channels. *Kcnk1* keeps the balance of the resting potential of the cellular membrane by controlling the outflow of potassium ions and has a vital role in the maintenance of neuron excitability. Paclitaxel can time‐dependently downregulate the expression of *Kcnk1* mRNA and protein in the DRG and trigeminal neurons.^16^, ^17^ Rescuing the downregulation can relieve mechanical allodynia and heat hyperalgesia,^17^ suggesting its potential involved in neuropathic pain. In the present study, similarly to in DRG, *Kcnk1* was found to be deceased in the spinal cord of neuropathic pain‐induced CCI surgery. This downregulation was attributed to the increased expression of spinal *miRNA‐26a* under the pain condition. Abolishing the increase of *miRNA‐26a* significantly restored the expression of *Kcnk1* in the spinal cord of mice with nerve injury, and the upregulation of *miRNA‐26a* led to the decrease of *Kcnk1* in the spinal cord of naïve mice, suggesting *Kcnk1* is a downstream gene regulated by *miRNA‐26a*. In line with this conclusion, the pain hypersensitivity induced by the *miRNA‐26a* increase was blocked or prevented by the *Kcnk1* upregulation. Interestingly, spinal *Kcnk1*, as a potassium ion responsible for the maintenance of resting potential in a spinal neuron, is demonstrated to be involved in the regulation of neuropathic pain. To our knowledge, it is the first evidence for *Kcnk1* associating with chronic pain at the spinal level. Additionally, given that the high conservation of *miRNA‐26a* binding to 3′UTR of *Kcnk1* among mammals such as humans, chimp, mice, and rats, our study may provide novel insight into the clinical treatment of neuropathic pain. In summary, we demonstrated that rescuing *ciRNA‐Kat6b* downregulation in the injured spinal cord mitigated neuropathic pain without affecting basal pain and locomotor function. Given that *ciRNA‐Kat6b* is specifically expressed in the nervous system tissues, developing drugs, or virus vehicles that increase its expression may selectively affect the nervous system without impacting peripheral tissues, and produce an anti‐nociception with no side effect on peripheral tissues compared to currently available treatments. Thus, *ciRNA‐Kat6b* may be a promising target for neuropathic pain management.

## AUTHOR CONTRIBUTIONS

Z. P. designed research; M. Z., L. X., Q.‐Q. L., R.‐N. W., M.‐L. S., Q. Z., L.‐Y. H., and Z.‐Y. X. performed research; Z. P., Q.‐H. W., Y. L., and H.‐J. W. analyzed data; M. Z. and Z. P. wrote the paper.

## CONFLICT OF INTEREST STATEMENT

The authors declare that there is no conflict of interests regarding the publication of this paper.

## Supporting information


Table S1

Figures S1

Figures S2

Figures S3

Appendix S1

## Data Availability

The data that support the findings of this study are available from the corresponding author upon reasonable request.

## References

[1] Cavalli E , Mammana S , Nicoletti F , Bramanti P , Mazzon E . The neuropathic pain: an overview of the current treatment and future therapeutic approaches. Int J Immunopathol Pharmacol. 2019;33:2058738419838383. 30900486 10.1177/2058738419838383 PMC6431761

[2] Gilron I , Baron R , Jensen T . Neuropathic pain: principles of diagnosis and treatment. Mayo Clin Proc. 2015;90(4):532‐545. 25841257 10.1016/j.mayocp.2015.01.018

[3] Baron R , Binder A , Wasner G . Neuropathic pain: diagnosis, pathophysiological mechanisms, and treatment. Lancet Neurol. 2010;9(8):807‐819. 20650402 10.1016/S1474-4422(10)70143-5

[4] Pan Z , Zhu LJ , Li YQ , et al. Epigenetic modification of spinal miR‐219 expression regulates chronic inflammation pain by targeting CaMKIIgamma. J Neurosci. 2014;34(29):9476‐9483. 25031391 10.1523/JNEUROSCI.5346-13.2014 PMC6608325

[5] Pan Z , Zhang M , Ma T , et al. Hydroxymethylation of microRNA‐365‐3p regulates nociceptive behaviors via Kcnh2. J Neurosci. 2016;36(9):2769‐2781. 26937014 10.1523/JNEUROSCI.3474-15.2016 PMC6604871

[6] Pan Z , Xue ZY , Li GF , et al. DNA Hydroxymethylation by ten‐eleven translocation Methylcytosine dioxygenase 1 and 3 regulates nociceptive sensitization in a chronic inflammatory pain model. Anesthesiology. 2017;127(1):147‐163. 28437360 10.1097/ALN.0000000000001632

[7] Pan Z , Shan Q , Gu P , et al. miRNA‐23a/CXCR4 regulates neuropathic pain via directly targeting TXNIP/NLRP3 inflammasome axis. J Neuroinflammation. 2018;15(1):29. 29386025 10.1186/s12974-018-1073-0 PMC5791181

[8] Pan Z , Li GF , Sun ML , et al. MicroRNA‐1224 splicing CircularRNA‐Filip1l in an Ago2‐dependent manner regulates chronic inflammatory pain via targeting Ubr5. J Neurosci. 2019;39(11):2125‐2143. 30651325 10.1523/JNEUROSCI.1631-18.2018 PMC6507086

[9] Pan Z , Zhang Q , Liu X , et al. Methyltransferase‐like 3 contributes to inflammatory pain by targeting TET1 in YTHDF2‐dependent manner. Pain. 2021;162(7):1960‐1976. 34130310 10.1097/j.pain.0000000000002218

[10] Kim WR , Park EG , Lee DH , Lee YJ , Bae WH , Kim HS . The tumorigenic role of circular RNA‐MicroRNA Axis in cancer. Int J Mol Sci. 2023;24(3):3050. 36769372 10.3390/ijms24033050 PMC9917898

[11] Najafi S , Aghaei Zarch SM , Majidpoor J , et al. Recent insights into the roles of circular RNAs in human brain development and neurologic diseases. Int J Biol Macromol. 2023;225:1038‐1048. 36410538 10.1016/j.ijbiomac.2022.11.166

[12] Pollema‐Mays SL , Centeno MV , Ashford CJ , Apkarian AV , Martina M . Expression of background potassium channels in rat DRG is cell‐specific and down‐regulated in a neuropathic pain model. Mol Cell Neurosci. 2013;57:1‐9. 23994814 10.1016/j.mcn.2013.08.002 PMC3842394

[13] Tsantoulas C . Emerging potassium channel targets for the treatment of pain. Curr Opin Support Palliat Care. 2015;9(2):147‐154. 25872119 10.1097/SPC.0000000000000131

[14] Lv YY , Wang H , Fan HT , Xu T , Xin WJ , Guo RX . SUMOylation of Kir7.1 participates in neuropathic pain through regulating its membrane expression in spinal cord neurons. CNS Neurosci Ther. 2022;28(8):1259‐1267. 35633059 10.1111/cns.13871 PMC9253747

[15] Blankenship ML , Coyle DE , Baccei ML . Transcriptional expression of voltage‐gated Na(+) and voltage‐independent K(+) channels in the developing rat superficial dorsal horn. Neuroscience. 2013;231:305‐314. 23219908 10.1016/j.neuroscience.2012.11.053 PMC3563431

[16] Mannerak MA , Lashkarivand A , Eide PK . Trigeminal neuralgia and genetics: a systematic review. Mol Pain. 2021;17:17448069211016139. 34000891 10.1177/17448069211016139 PMC8135221

[17] Mao Q , Wu S , Gu X , et al. DNMT3a‐triggered downregulation of K2p 1.1 gene in primary sensory neurons contributes to paclitaxel‐induced neuropathic pain. Int J Cancer. 2019;145(8):2122‐2134. 30684388 10.1002/ijc.32155 PMC6660426

[18] Pan Z , Du S , Wang K , et al. Downregulation of a dorsal root ganglion‐specifically enriched Long noncoding RNA is required for neuropathic pain by negatively regulating RALY‐triggered Ehmt2 expression. Advanced Science. 2021;8(13):e2004515. 34383386 10.1002/advs.202004515 PMC8356248

[19] Yang JX , Hua L , Li YQ , et al. Caveolin‐1 in the anterior cingulate cortex modulates chronic neuropathic pain via regulation of NMDA receptor 2B subunit. J Neurosci. 2015;35(1):36‐52. 25568101 10.1523/JNEUROSCI.1161-14.2015 PMC6605256

[20] Li XY , Toyoda H . Role of leak potassium channels in pain signaling. Brain Res Bull. 2015;119(Pt A):73‐79. 26321392 10.1016/j.brainresbull.2015.08.007

[21] Kristensen LS , Andersen MS , Stagsted LVW , Ebbesen KK , Hansen TB , Kjems J . The biogenesis, biology and characterization of circular RNAs. Nat Rev Genet. 2019;20(11):675‐691. 31395983 10.1038/s41576-019-0158-7

[22] Memczak S , Jens M , Elefsinioti A , et al. Circular RNAs are a large class of animal RNAs with regulatory potency. Nature. 2013;495(7441):333‐338. 23446348 10.1038/nature11928

[23] Rybak‐Wolf A , Stottmeister C , Glazar P , et al. Circular RNAs in the mammalian brain are highly abundant, conserved, and dynamically expressed. Mol Cell. 2015;58(5):870‐885. 25921068 10.1016/j.molcel.2015.03.027

[24] Tay Y , Rinn J , Pandolfi PP . The multilayered complexity of ceRNA crosstalk and competition. Nature. 2014;505(7483):344‐352. 24429633 10.1038/nature12986 PMC4113481

[25] Zhang ZJ , Guo JS , Li SS , et al. TLR8 and its endogenous ligand miR‐21 contribute to neuropathic pain in murine DRG. J Exp Med. 2018;215(12):3019‐3037. 30455267 10.1084/jem.20180800 PMC6279408

[26] Jiang BC , Cao DL , Zhang X , et al. CXCL13 drives spinal astrocyte activation and neuropathic pain via CXCR5. J Clin Invest. 2016;126(2):745‐761. 26752644 10.1172/JCI81950 PMC4731172

[27] Li H , Wan HQ , Zhao HJ , Luan SX , Zhang CG . Identification of candidate genes and miRNAs associated with neuropathic pain induced by spared nerve injury. Int J Mol Med. 2019;44(4):1205‐1218. 31432094 10.3892/ijmm.2019.4305 PMC6713433

[28] Cheng Q , Tang L , Wang Y . Regulatory role of miRNA‐26a in neonatal sepsis. Exp Ther Med. 2018;16(6):4836‐4842. 30542439 10.3892/etm.2018.6779 PMC6257428

[29] Liang Z , Chi YJ , Lin GQ , Luo SH , Jiang QY , Chen YK . MiRNA‐26a promotes angiogenesis in a rat model of cerebral infarction via PI3K/AKT and MAPK/ERK pathway. Eur Rev Med Pharmacol Sci. 2018;22(11):3485‐3492. 29917203 10.26355/eurrev_201806_15175

[30] Jing R , Zhong QQ , Long TY , Pan W , Qian ZX . Downregulated miRNA‐26a‐5p induces the apoptosis of endothelial cells in coronary heart disease by inhibiting PI3K/AKT pathway. Eur Rev Med Pharmacol Sci. 2019;23(11):4940‐4947. 31210329 10.26355/eurrev_201906_18084

[31] Chen J , Xu Y , Tao L , et al. MiRNA‐26a contributes to the Acquisition of Malignant Behaviors of Doctaxel‐resistant lung adenocarcinoma cells through targeting EZH2. Cell Physiol Biochem. 2017;41(2):583‐597. 28214878 10.1159/000457879

