# Supplementary figures and images for: Downregulation of ciRNA‐ Kat6b in dorsal spinal horn is required for neuropathic pain by regulating Kcnk1 in miRNA‐26a‐dependent manner
Source: CNS Neurosci Ther. 2023 May 5;29(10):2955–71. doi: 10.1111/cns.14235 (PMC10493661; doi:10.1111/cns.14235)

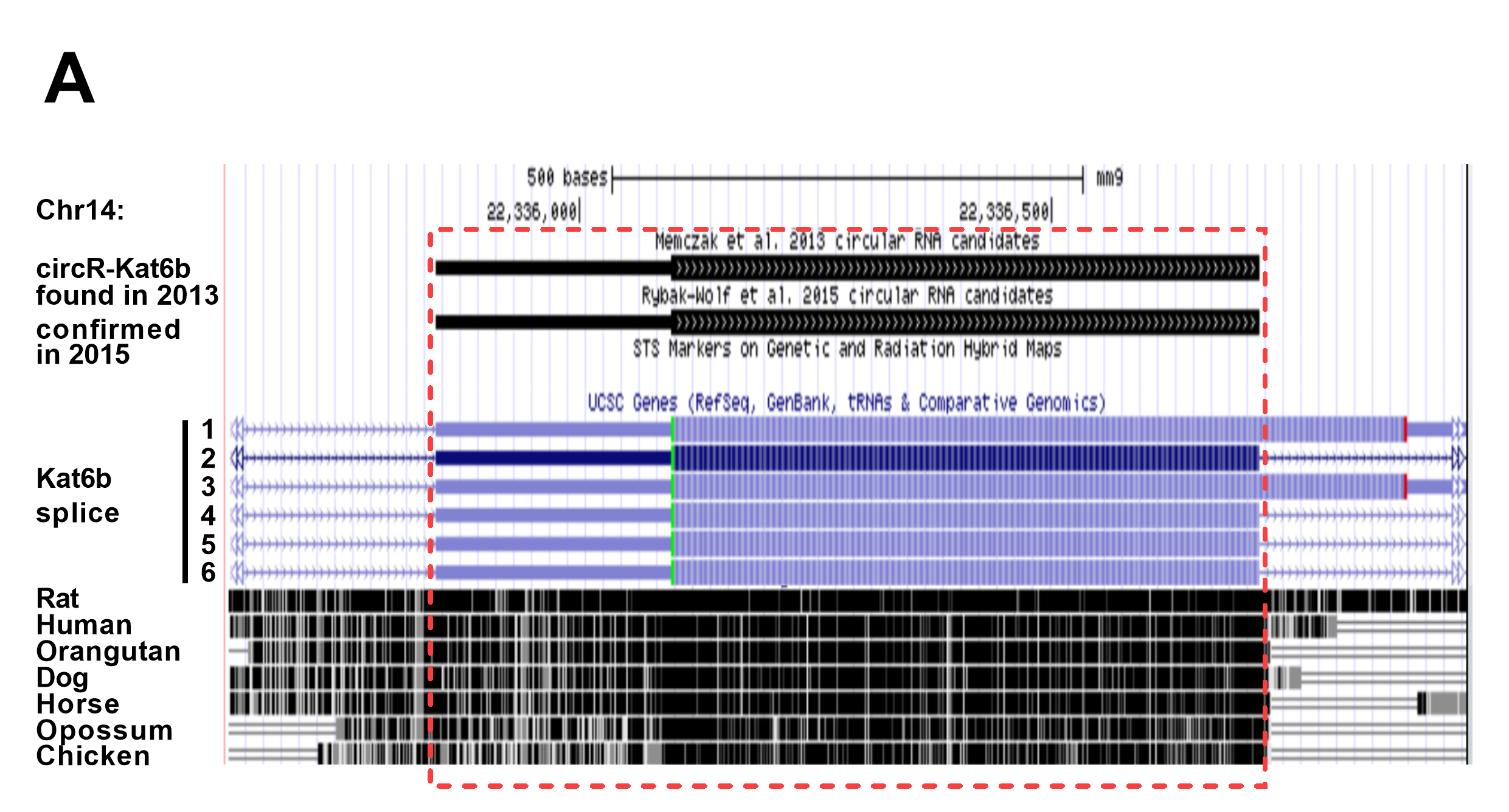

Supplement: Supplementary file 2 — Figures S1 [file CNS-29-2955-s001.tif]

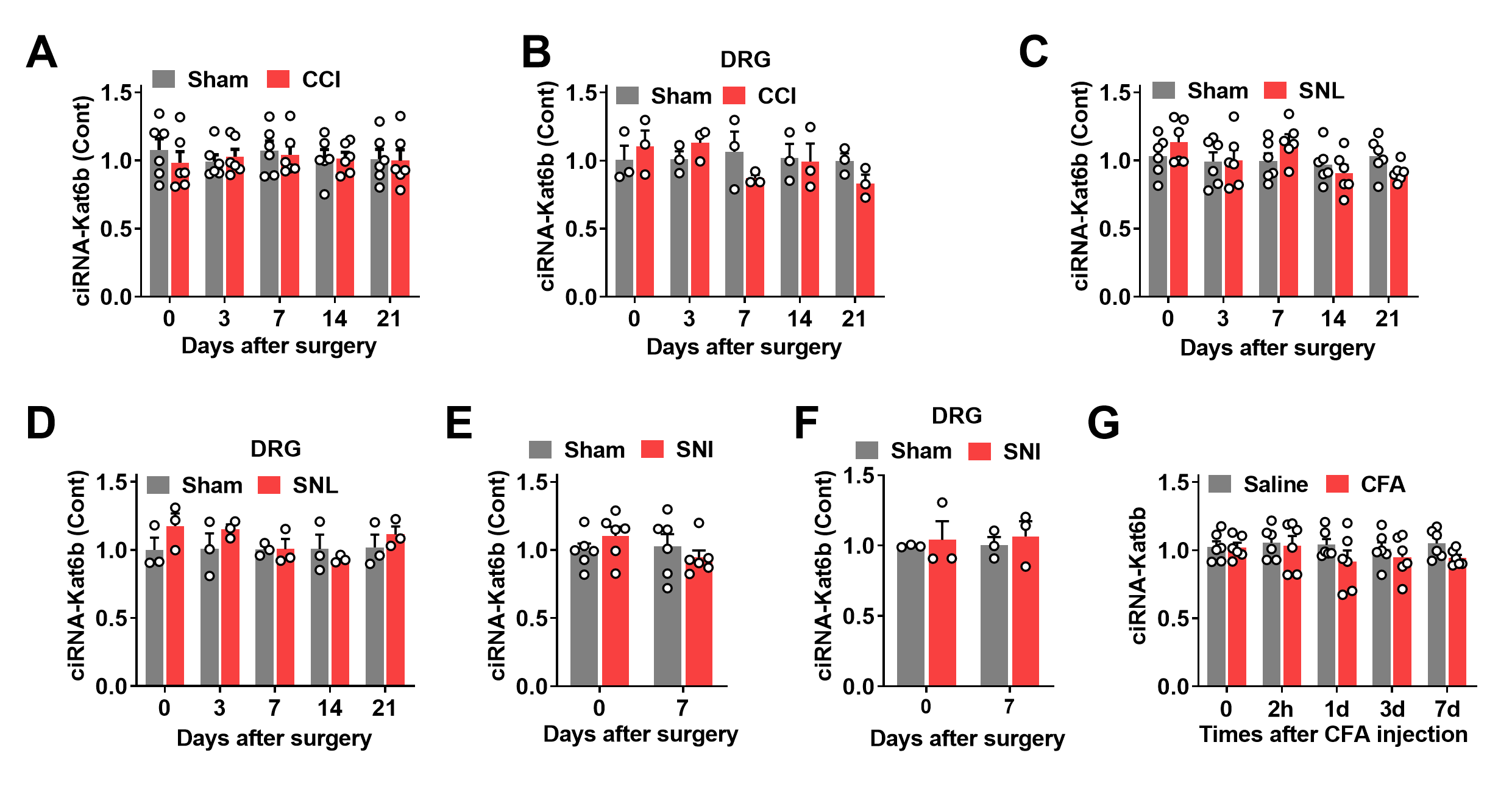

Supplement: Supplementary file 3 — Figures S2 [file CNS-29-2955-s002.tif]

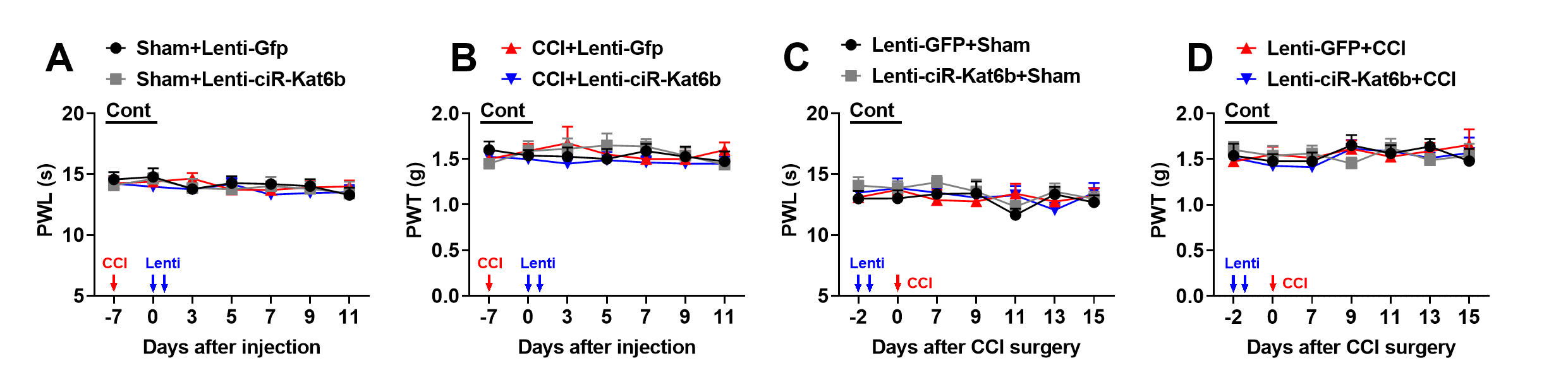

Supplement: Supplementary file 4 — Figures S3 [file CNS-29-2955-s005.tif]
