# Supplementary material for: Downregulation of ciRNA‐ Kat6b in dorsal spinal horn is required for neuropathic pain by regulating Kcnk1 in miRNA‐26a‐dependent manner
Source: CNS Neurosci Ther. 2023 May 5;29(10):2955–71. doi: 10.1111/cns.14235 (PMC10493661; doi:10.1111/cns.14235)
Supplement: Supplementary file 5 — Appendix S1 [file CNS-29-2955-s004.pdf]

Note: The gel image in the square was used in Figures in this study.

Figure 1B

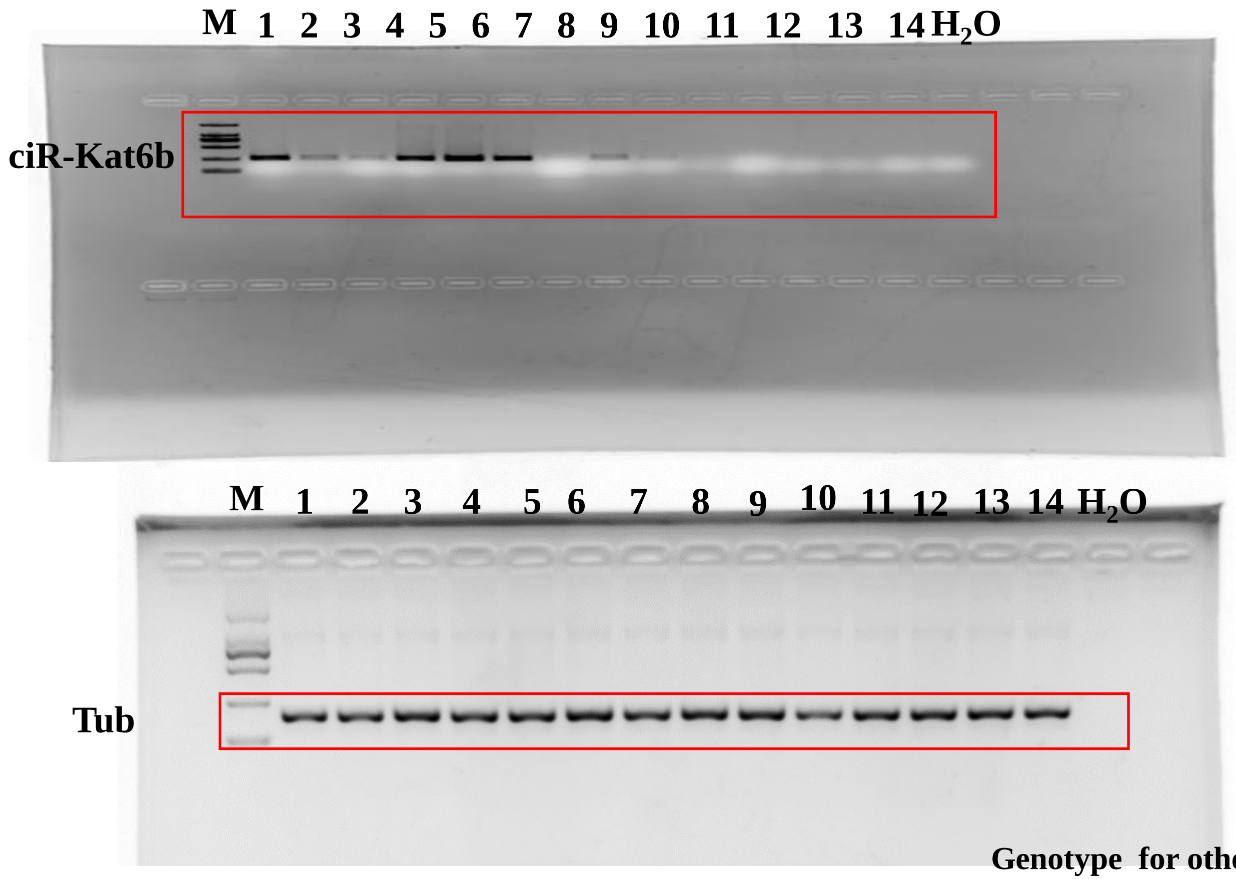

Figure 1D

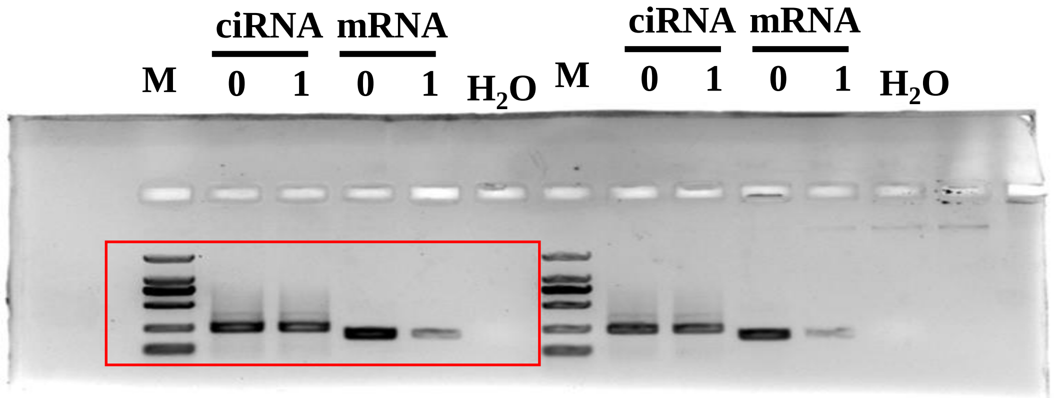

Figure 1F

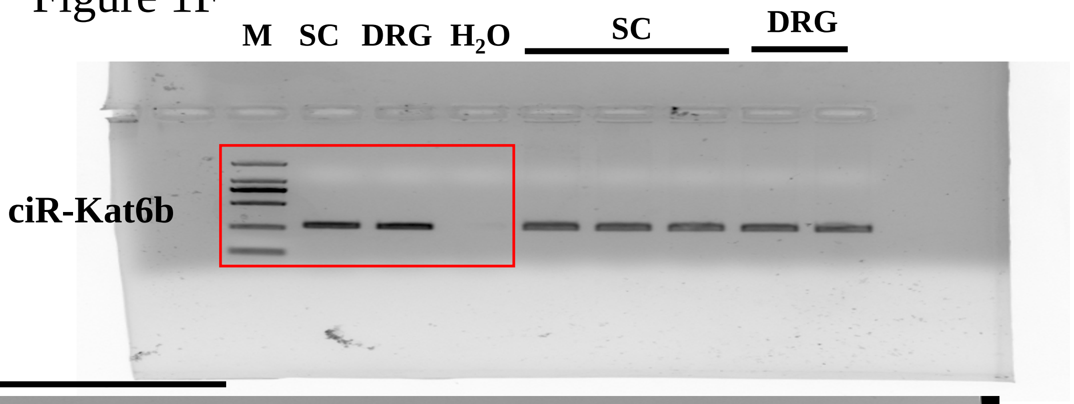

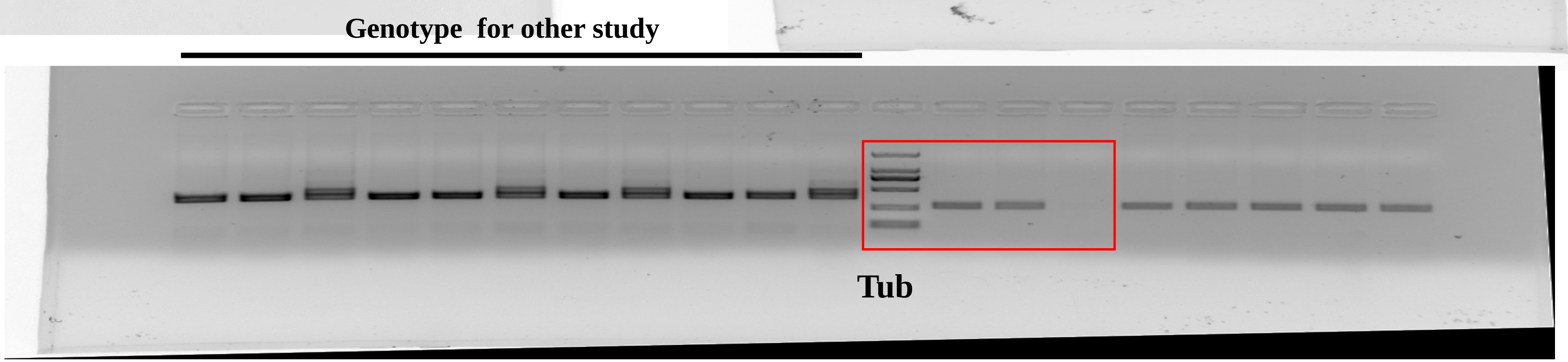

Figure 4B

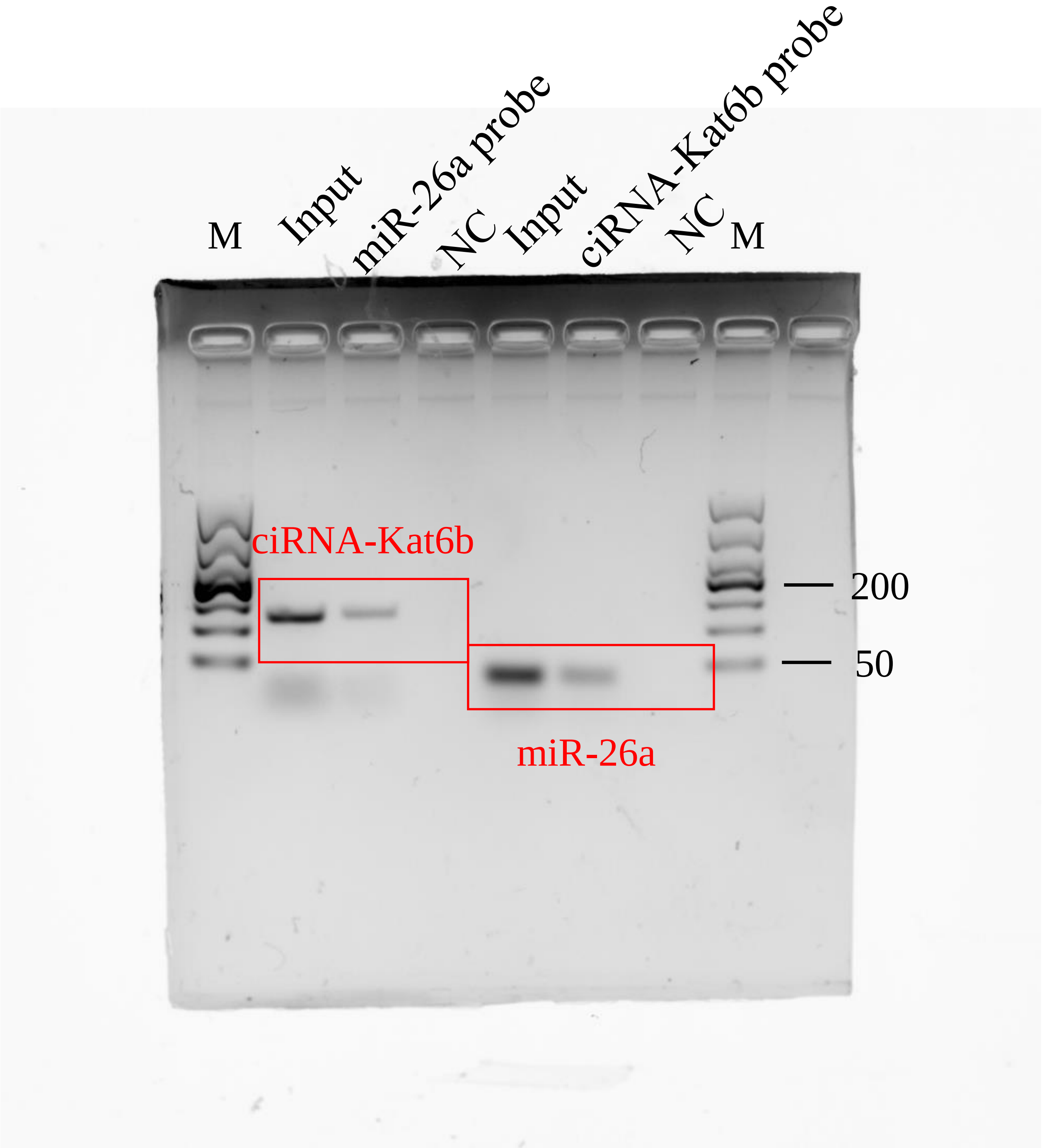

M
Input
miR-26a probe
NC
Input
ciRNA-Kat6b probe
NC
M
ciRNA-Kat6b
200
50
miR-26a

Figure 6D

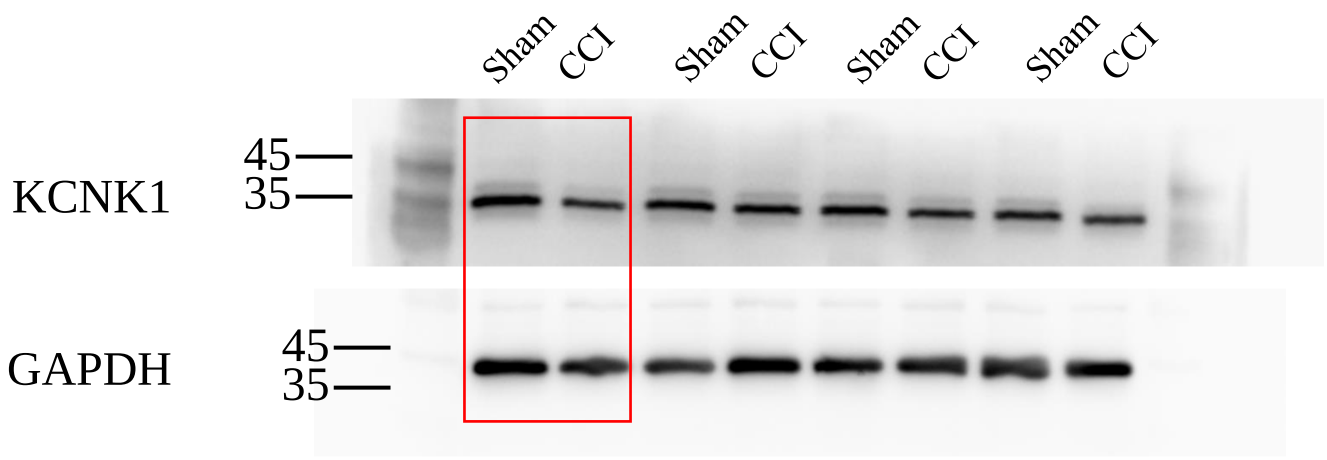

Figure 6E

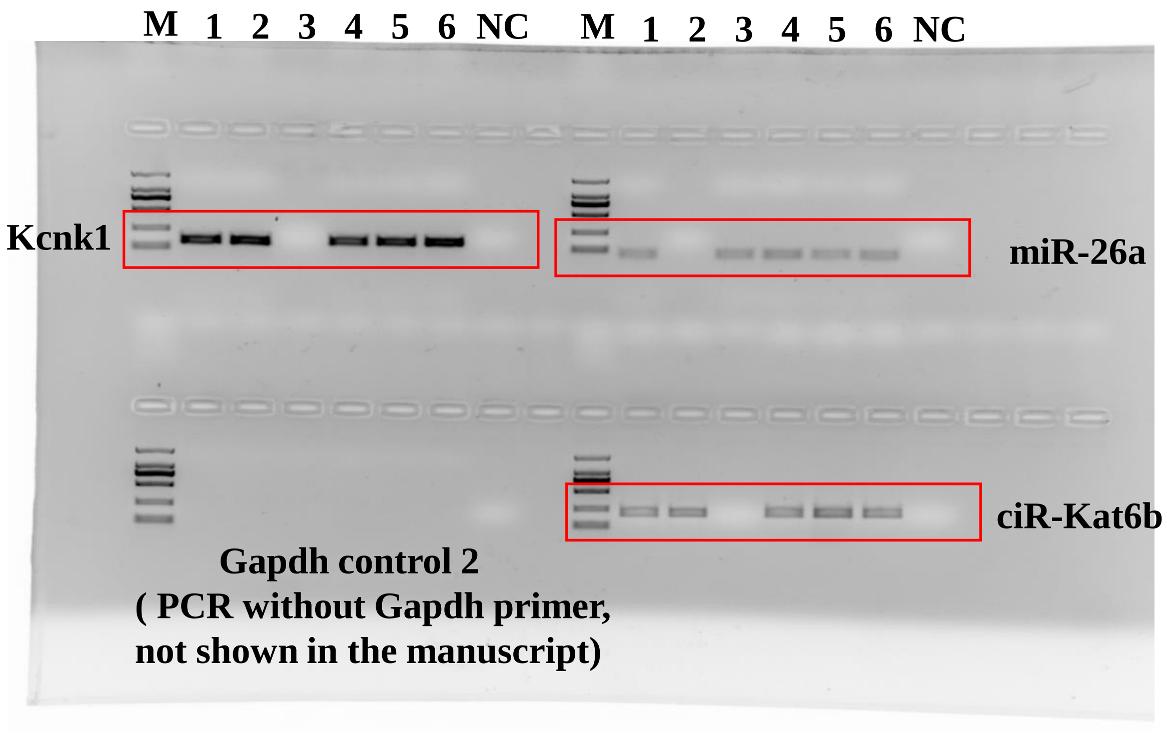
M 1 2 3 4 5 6 NC M 1 2 3 4 5 6 NC
Kcnk1
miR-26a
ciR-Kat6b
Gapdh control 2
(PCR without Gapdh primer,
not shown in the manuscript)

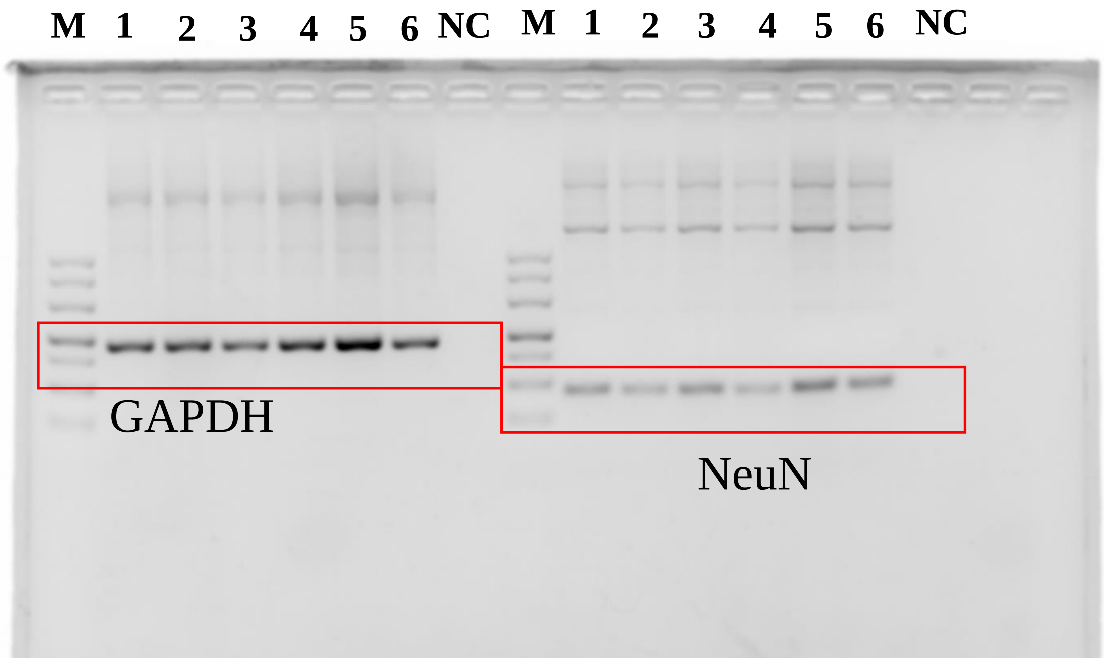
M 1 2 3 4 5 6 NC M 1 2 3 4 5 6 NC
GAPDH
NeuN

Figure 6H

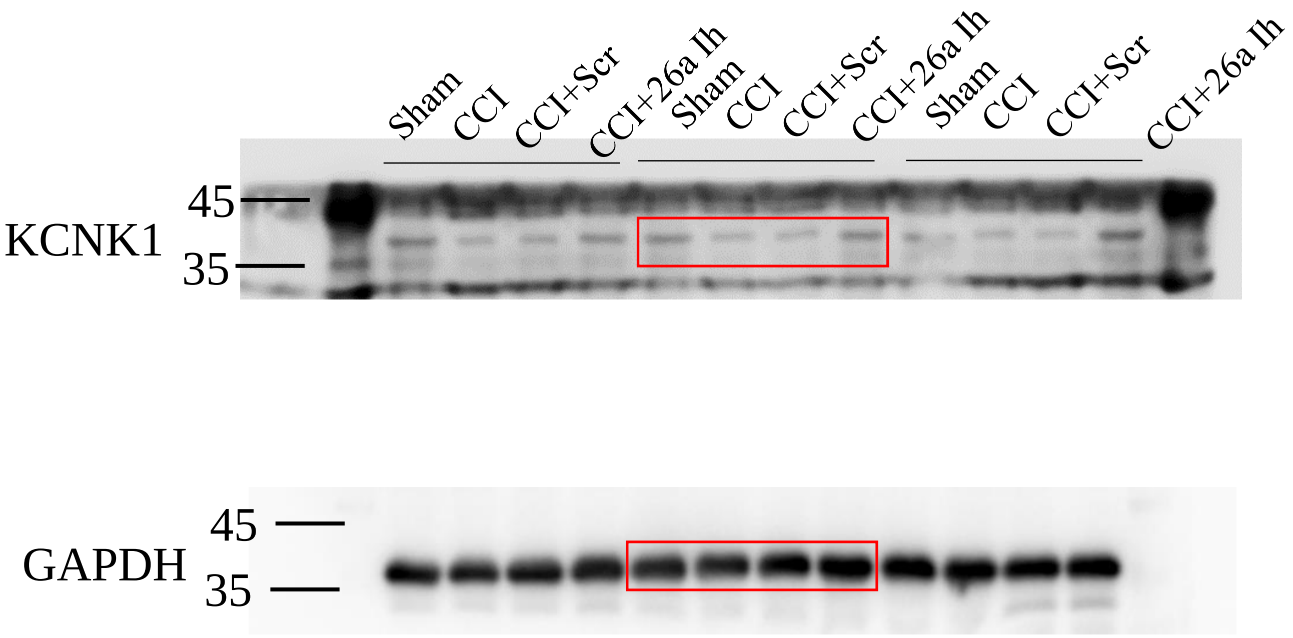
Sham
CCI
CCI+Scr
CCI+26a Ih
Sham
CCI
CCI+Scr
CCI+26a Ih
Sham
CCI
CCI+Scr
CCI+26a Ih
KCNK1
45
35
GAPDH
45
35

Figure 6J

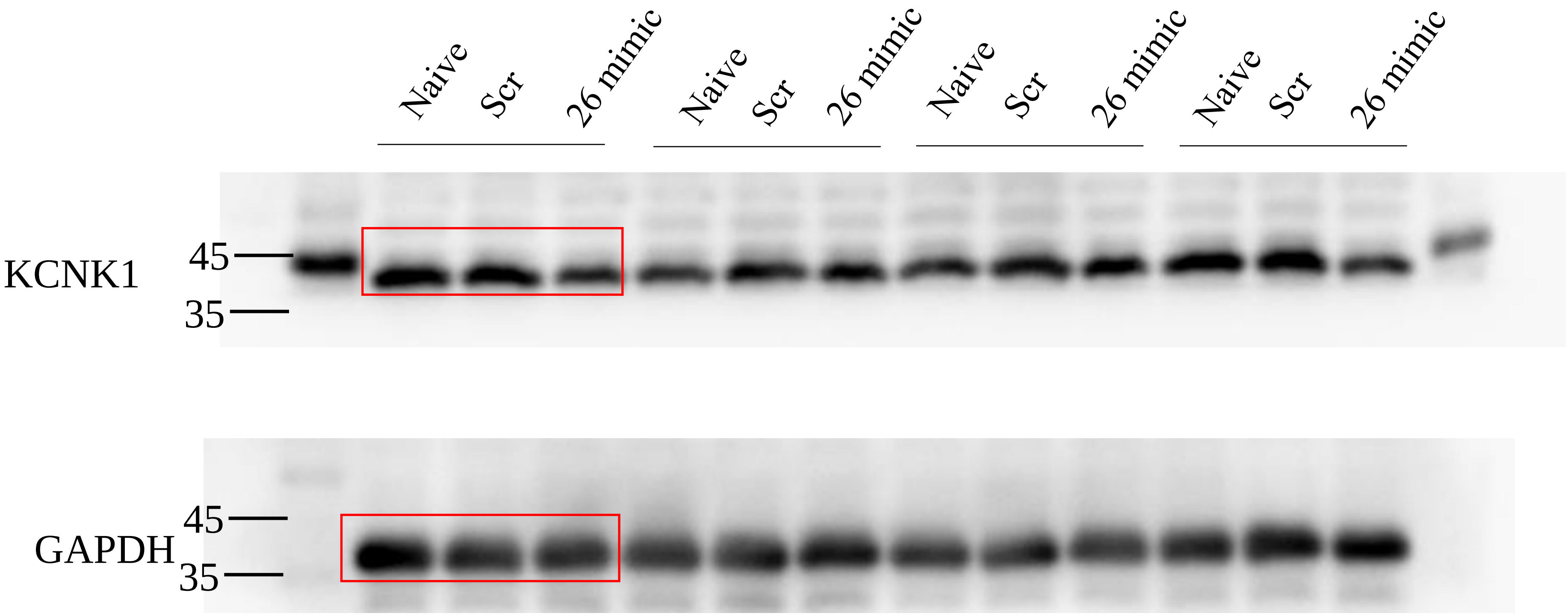

Naive
Scr
26 mimic
Naive
Scr
26 mimic
Naive
Scr
26 mimic
Naive
Scr
26 mimic
KCNK1
45
35
GAPDH
45
35

Figure 7A

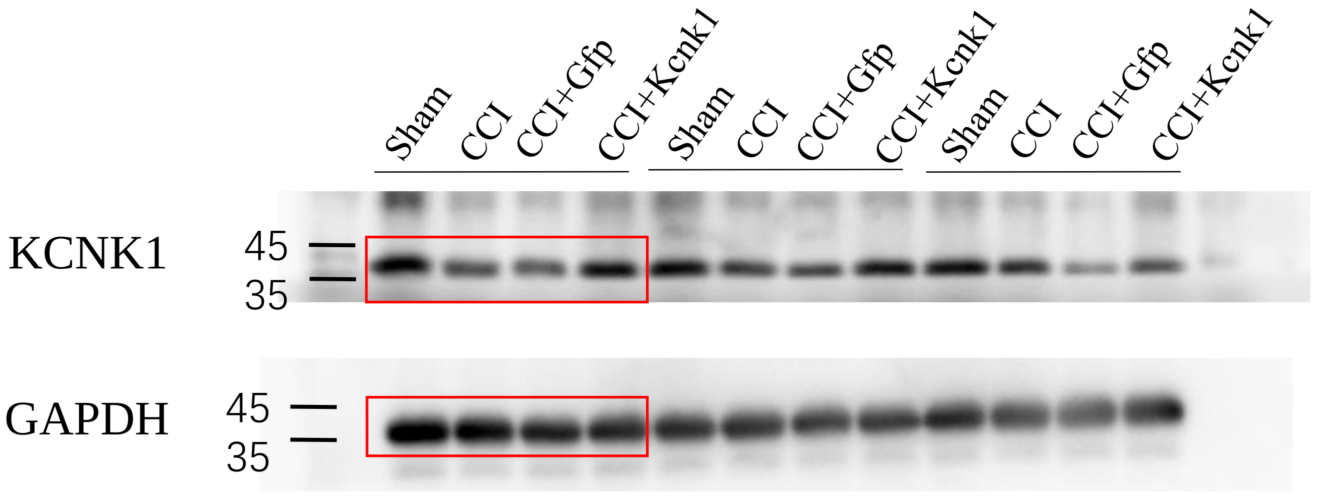
Sham
CCI
CCI+Gfp
CCI+Kcnk1
Sham
CCI
CCI+Gfp
CCI+Kcnk1
Sham
CCI
CCI+Gfp
CCI+Kcnk1
KCNK1
45
35
GAPDH
45
35

Figure 7D

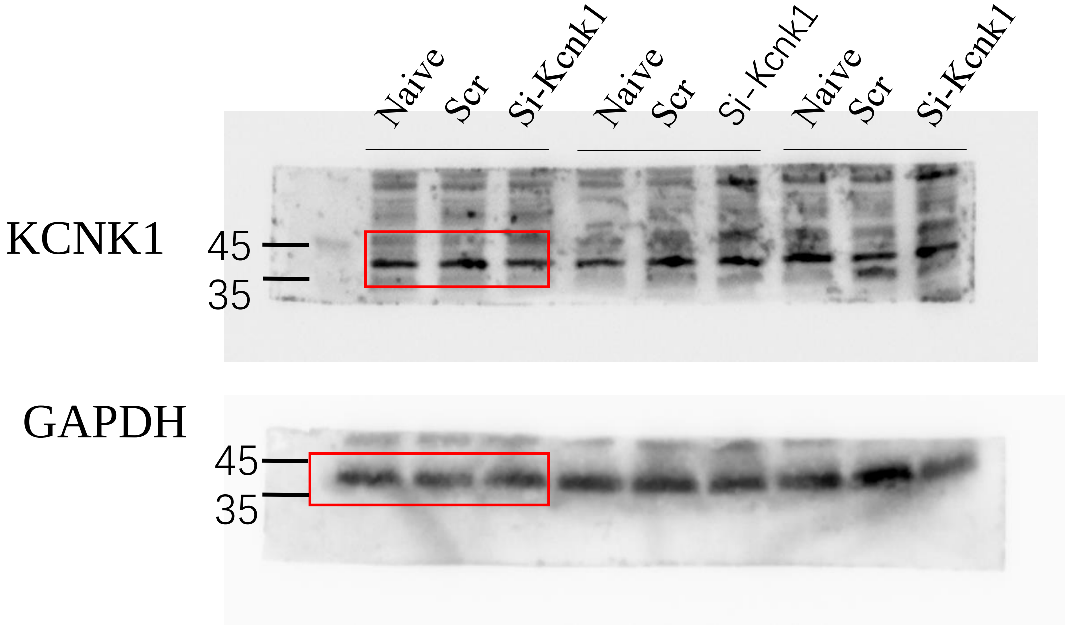

Naive
Scr
Si-Kcnk1
Naive
Scr
Si-Kcnk1
Naive
Scr
Si-Kcnk1
KCNK1
45
35
GAPDH
45
35
